# Genomic and immunogenomic analysis of three prognostic signature genes in LUAD

**DOI:** 10.1186/s12859-023-05137-y

**Published:** 2023-01-17

**Authors:** Hai-Ming Feng, Ye Zhao, Wei-Jian Yan, Bin Li

**Affiliations:** 1 Department of Thoracic Surgery, Lanzhou University Second Hospital, Lanzhou University Second Clinical Medical College, 82 Cuiyingmen, Chengguan District, Lanzhou, 730030 Gansu People’s Republic of China; 2 Department of Radiotherapy, Gansu Provincial People’s Hospital, Lanzhou City, 730030 China

**Keywords:** Lung adenocarcinoma, Immunotherapy, Prognostic analysis, Tumor microenvironment

## Abstract

**Background:**

Searching for immunotherapy-related markers is an important research content to screen for target populations suitable for immunotherapy. Prognosis-related genes in early stage lung cancer may also affect the tumor immune microenvironment, which in turn affects immunotherapy.

**Results:**

We analyzed the differential genes affecting lung cancer patients receiving immunotherapy through the Cancer Treatment Response gene signature DataBase (CTR-DB), and set a threshold to obtain a total of 176 differential genes between response and non-response to immunotherapy. Functional enrichment analysis found that these differential genes were mainly involved in immune regulation-related pathways. The early-stage lung adenocarcinoma (LUAD) prognostic model was constructed through the cancer genome atlas (TCGA) database, and three target genes (*MMP12*, *NFE2*, HOXC8) were screened to calculate the risk score of early-stage LUAD. The receiver operating characteristic (ROC) curve indicated that the model had good prognostic value, and the validation set (GSE50081, GSE11969 and GSE42127) from the gene expression omnibus (GEO) analysis indicated that the model had good stability, and the risk score was correlated with immune infiltrations to varying degrees. Multi-type survival analysis and immune infiltration analysis revealed that the transcriptome, methylation and the copy number variation (CNV) levels of the three genes were correlated with patient prognosis and some tumor microenvironment (TME) components. Drug sensitivity analysis found that the three genes may affect some anti-tumor drugs. The mRNA expression of immune checkpoint-related genes showed significant differences between the high and low group of the three genes, and there may be a mutual regulatory network between immune checkpoint-related genes and target genes. Tumor immune dysfunction and exclusion (TIDE) analysis found that three genes were associated with immunotherapy response and maybe the potential predictors to immunotherapy, consistent with the CTR-DB database analysis.

**Conclusions:**

From the perspective of data mining, this study suggests that *MMP12*, *NFE2*, and HOXC8 may be involved in tumor immune regulation and affect immunotherapy. They are expected to become markers of immunotherapy and are worthy of further experimental research.

**Supplementary Information:**

The online version contains supplementary material available at 10.1186/s12859-023-05137-y.

## Introduction

Lung cancer is the malignant tumor with the second highest incidence and the highest mortality in the world [1, 2]. According to the GLOBOCAN analysis report of the global tumor epidemiological statistics in 2020, the number of new cases of lung cancer worldwide reached 2.207 million, second only to breast cancer; the number of deaths reached 1.796 million, ranking first among all cancer types. LUAD is the most common pathological type of non-small cell lung cancer (NSCLC). For driver gene-negative advanced NSCLC, the median progression free survival of traditional platinum-based doublet chemotherapy is only 4–6 months, and the median overall survival is only 10–12 months [3], and immunotherapy can bring survival benefit to driver gene-negative advanced NSCLC. Researchers [4] predicted that the advent of immunotherapy will further improve the survival outcomes of lung cancer patients, especially for advanced NSCLC with negative driver gene mutations. The food and drug administration (FDA) approved the first immune checkpoint inhibitors (ICIs) for the treatment of lung cancer in 2015. Over the past few years, the number of ICIs approved and applied in the clinic has gradually increased, and a few other ICIs are currently in clinical development [5], and peptides and small peptides targeting programmed cell death ligand 1 (*PD-L1*) have also been designed. molecules whose purpose is to block checkpoints and activate T-cell-based immunotherapy [6]. For patients with advanced NSCLC with tumor proportional score(TPS) of *PD-L1* ≥ 1%, immune monotherapy can significantly improve the progression-free survival (PFS) and overall survival (OS) of patients compared with chemotherapy, especially for patients with TPS ≥ 50%, while immunotherapy combined with chemotherapy significantly prolonged PFS and OS of patients with *PD-L1* negative and driver-gene-negative advanced non-squamous NSCLC [7–10]. Positive responses to immunotherapy often rely on the interaction of tumor cells with immune regulation within the TME. The tumor microenvironment plays an important role in suppressing or enhancing immune responses. Understanding the interaction between immunotherapy and TME is not only the key to dissect the mechanism of action, but also of great significance to provide new methods for improving the efficacy of current immunotherapy [11, 12]. Since the main cell components that maintain the immunosuppressive microenvironment also play an anti-tumor role in the early stage of tumor progression, the immunotherapy strategy targeting TME can stimulate or restore the inherent anti-tumor ability of the immune system, reshape the positive TME, and produce a comprehensive response effect. Therefore, drug development for TME is also accelerating, including targeting hypoxia inducible factor-1 α, tumor matrix, angiogenesis and tumor related macrophages [13–15]. In addition, recent research on nano drug delivery systems based on the unique characteristics of TME is expected to enhance anti-tumor therapy [16]. Although ICIs have shown excellent efficacy in NSCLC, their efficacy varies widely, only a subset of patients, especially those with high *PD-L1* expression, benefit from long-term responses, and a large proportion of patients do not show obvious curative effect or drug resistance. For these reasons, it is necessary to combine the gene landscape of tumor immunotherapy to discover and search for potential molecules and mechanisms affecting immunotherapy, to screen target populations, and to guide individualized treatment. Obviously, even if no intervention is given after surgery for early-stage lung cancer, a good survival benefit can still be obtained. This is not only related to the biological characteristics of the tumor, but also the immune function may play a huge role in preventing tumor recurrence or distant metastasis. In recent years, machine learning and deep learning algorithms have been used to train many models represented by feature gene sets to predict the prognosis of NSCLC patients based on high-throughput gene expression data and survival data, including the short-term efficacy and long-term survival prediction of immunotherapy. However, the prediction effect is uneven and there is no unified measurement standard, so there are limitations in clinical transformation and popularization [17, 18]. Therefore, we tried to find differential genes that may affect the response to immunotherapy, construct target genes that have a significant impact on the prognosis of early-stage lung cancer, and then analyze the relationship between the multi-omics changes of these genes and the tumor microenvironment of all stages of LUAD.

## Materials and methods

### Immunotherapy response differential genes (ImTRDG)

**Fig. 1:**
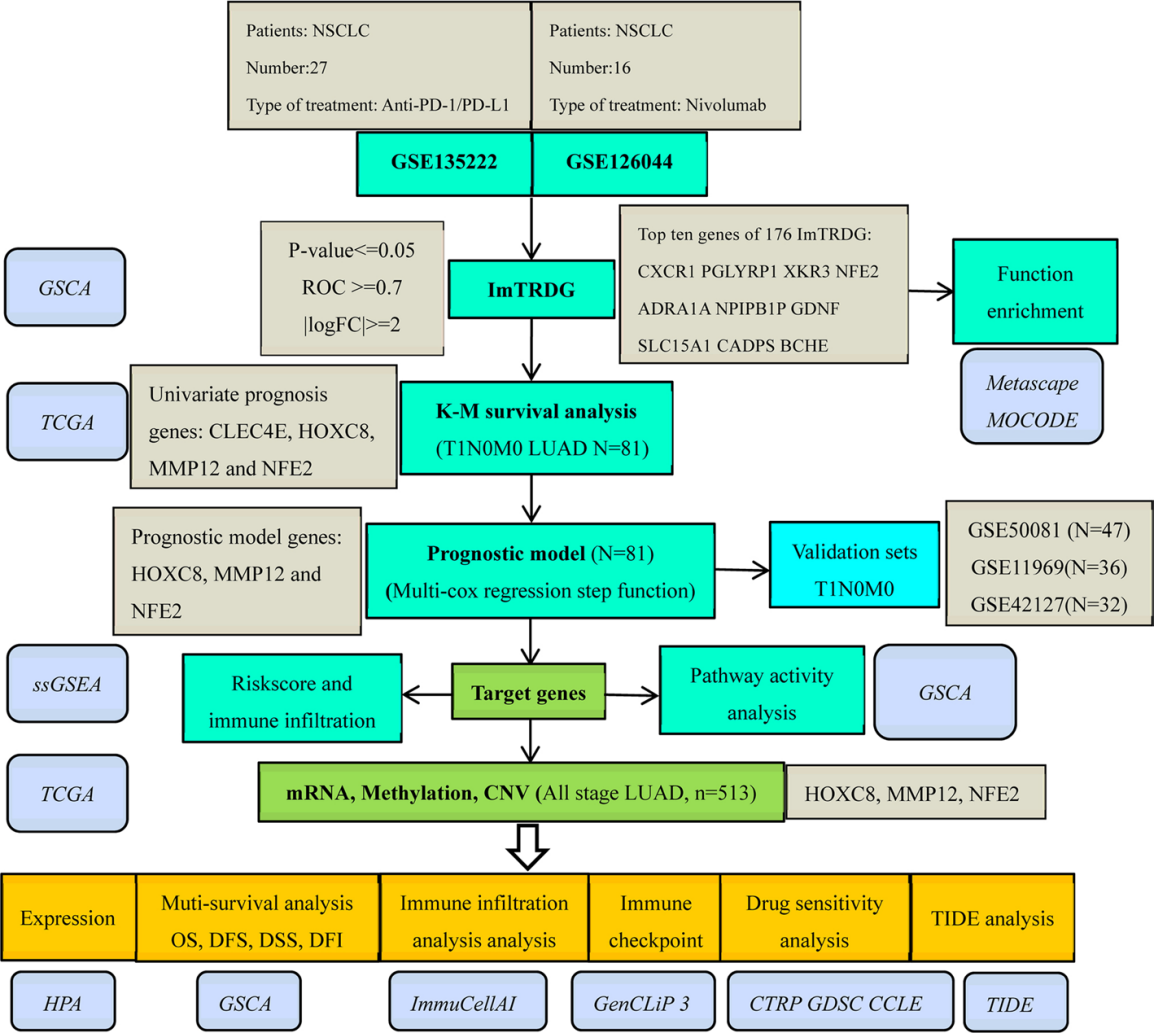
The overall process of research contents

The overall process of the article is shown in Fig. 1.

The mRNA and clinical data of NSCLC patients treated with anti-*PD-1/PD-L1* were collected through the CTR-DB (http://ctrdb.cloudna.cn/home) [19] website, including GSE135222 [20] and GSE126044 [21] data sets from the GEO database, according to the response to immunotherapy, they were divided into responder [CR (complete response) and PR (partial response)] and non-responder [SD (stable disease) and PD(progressive disease)], the patient responses in the CTR-DB calibrated in accordance to RECIST v1.1 criteria, and the differences in mRNA expression between responder and non-responder groups were analyzed and compared. Differential genes were screened by setting the threshold adjusted *P* value <  = 0.05 and |logFC|> = 2, and the target genes were determined according to the AUC (Area under roc Curve) value >  = 0.7.

### ImTRDG functional enrichment analysisc

The ImTRDG was imported into the Metascape website (https://metascape.org/) [22] for functional enrichment analysis and protein interaction analysis, and the Molecular Complex Detection (MOCODE) [23] algorithm was used to find dense PPI MOCODE (protein–protein interaction) components in the network and annotate them. In the network analysis, set min connection to 3, *p* value cutoff to 0.01, and min enrichment to 1.5. In the protein interaction network analysis, the reference database is PHYSICAL_CORE, the min network size is 3, and the max network size is 500.

### Univariate Cox regression analysis of ImTRDG

RNAseq data (FPKM; Fragments Per Kilobase of transcript per Million mapped reads) and corresponding clinical information for T1N0M0 stage LUAD were obtained from TCGA dataset (https://portal.gdc.com). The log-rank was used to test the Kaplan–Meier survival analysis to compare the difference in survival between the high and low expression group of ImTRDG genes. For KM curves, *p* values and hazard ratios (HR) with 95% confidence intervals (CI) were derived by log rank test and univariate cox regression. *p* < 0.05 was considered statistically significant.

### Prognosis signature establishment and immune infiltration analysis

After obtaining prognostic genes through univariate cox regression, First, perform iterative analysis through multi-factor cox regression analysis, and then select the optimal model to reduce dimensionality and build a prognostic model through the step function, The model is a risk-score formula containing multiple genes, each gene has a weight, a negative number means the gene is a protective gene, and a positive number means the gene is a risk gene, and the R software glmnet package was used for the above analysis. For Kaplan–Meier curves, *p* values and HR with 95% CI were obtained by log-rank test and univariate cox regression and time-ROC analysis was used to discriminate the accuracy of the prediction model. *p* < 0.05 was considered statistically significant. Finally, the stability of the model was verified using the GSE50081, GSE11969 and GSE42127datasets which were derived from the GEO database and contains the expression profiles and clinical data of 47, 33 and 32 T1N0M0 LUAD samples, respectively [24–26]. Then the immune infiltration scores of T1N0M0 LUAD samples were calculated by MCpcounter package of R program v4.0.3 [27], and the correlation between risk-score and individual immune infiltration component scores was analyzed. Spearman's correlation analysis was used to describe correlations between quantitative variables without a normal distribution. *P* value less than 0.05 was considered statistically significant.

### Prognostic models based on gene expression and clinical characteristics

After screening the characteristic genes of T1N0M01 LUAD by cox step model, combined with clinical characteristics, firstly, univariate and multivariate cox regression analysis. Each variable (P-value, HR and 95% CI) was displayed using a forest plot by the "forestplot" package. Based on the results of a multivariate cox proportional hazards analysis, a nomogram was built using the "rms" package to predict the year total recurrence rate. The nomogram provides a graphical result of these factors, and the prognostic time risk of an individual patient can be calculated by the points associated with each risk factor.

## Expression and compiled scores analysis

### Difference analysis

LUAD gene expression data were obtained from the TCGA database and GTEx database. Based on normalized RSEM (RNA-Seq by expectation maximization) mRNA expression, fold change was calculated by mean (tumor)/mean (normal), p-value was estimated by Wilcox tests and false discovery rate (FDR) was used to analyze differences between LUAD patients in whole samples. At the same time, the Human Protein Atlas (HPA) database (https://www.proteinatlas.org/) was used to search the immunohistochemical results of the target gene translation protein in LUAD and normal samples.

### Survival prognostic analysis

Merged mRNA expression and clinical survival data by sample barcode, median mRNA value was used to divide tumor samples into high and low expression groups. Then, we use R package survival to fit survival time and survival status within two groups. Cox proportional-hazards model and log rank tests were performed for every gene in LUAD. Survival types including overall survival (OS), progression free survival (PFS), disease specific survival (DSS), and disease free interval (DFI).

### Potential effects of gene mRNA on pathway activity

Reverse phase protein array (RPPA) data from (The Cancer Proteome Atlas database) were used to calculate pathway activity score for TCGA LUAD samples. RPPA is a high-throughput antibody-based technique with the procedures similar to that of western blots. Proteins are extracted from tumor tissue or cultured cells, denatured by SDS, printed on nitrocellulose-coated slides followed by antibody probe. Expression and pathway activity can estimate the difference of genes expression between pathway activity groups (activation and inhibition), which defined by median pathway scores. The Gene Set Cancer Analysis (GSCA) [28] pathway included are: *TSC/mTOR*, *RTK*, *RAS/MAPK*, *PI3K/AKT*, Hormone *ER*, Hormone *AR*, *EMT*, DNA Damage Response, Cell Cycle, Apoptosis pathways. They are all well-studied cancer related pathways. RPPA data were median-centered and normalized by standard deviation across all samples for each component to obtain the relative protein level. The pathway score is then the sum of the relative protein level of all positive regulatory components minus that of negative regulatory components in a particular pathway. Samples were divided into 2 groups (high and low) by median gene expression, the difference of pathway activity score (PAS) between groups is defined by student t test, p value was adjusted by FDR, FDR <  = 0.05 is considered as significant. When PAS (Gene A High expression) > PAS (Gene A Low expression), we consider gene A may have an activate effect to a pathway, otherwise have an inhibitory effect to a pathway. In addition, according to the ssGSEA (single sample gene set enrichment analys) algorithm, the enrichment score of each sample on each pathway is calculated in turn, so as to obtain the relationship between the sample and the pathway. By calculating the correlation between the gene expression and the pathway score, the relationship between the gene and the pathway can be obtained [29, 30].

### CNV and methylation analysis of target genes

CNV data of LUAD samples were downloaded from TCGA database, and were processed through GISTICS2.0, which attempts to identify significantly altered regions of amplification or deletion across sets of patients. According to the GISTIC score derived from GISTIC, CNV was classified into homozygous deletion, heterozygous deletion, heterozygous amplification and homozygous amplification. The mRNA expression data and CNV raw data were merged by TCGA barcode. CNV data and clinical survival data were merged by sample barcode. The samples were divided into WT, Amp. and Dele. groups. R survival package was used to fit survival time and survival status within groups. Log rank tests were performed to test the survival difference between groups. Finally, we integrate the CNV of a single target gene and call it a gene set CNV, the gene set CNV represents the integrated CNV status of target gene set for each sample. A sample is classified into Amp. or Dele. group. If all genes in inputted gene set have no CNV in a sample, this sample is classified into WT group. The association of gene set CNVs with survival prognosis was then analyzed.

LUAD Illumina Human Methylation 450 k data were downloaded from TCGA database, Methylation data and clinical survival data were combined by sample barcodes. The median methylation was used to classify tumor samples into hypermethylated and hypomethylated groups. The cox proportional-hazards model was constructed to obtain the hazard ratio of the hypermethylated group to the hypomethylated group. A log rank test was performed to test whether the difference in survival between groups was statistically significant.

### Immune infiltration analysis

The infiltration of 24 immune cells was assessed by ImmuCellAI database, and the association between gene mRNA expression, gene CNVs (copy number variations), gene methylation and gene set CNVs and immune cell infiltration was estimated [31, 32].

### Drug sensitivity analysis

We collected the IC50s and their corresponding mRNA gene expressions of 481 small molecules in 1001 cell lines from the Genomics of Therapeutics Response Portal (CTRP) [33]. Also Genomics of Drug Sensitivity in Cancer (GDSC) [34] contained the IC50 of 265 small molecules in 860 cell lines, the IC50 corresponding mRNA gene expression from mRNA expression data and drug sensitivity data were combined. Pearson correlation analysis was performed to obtain the correlation between gene mRNA expression and drug IC50. At the same time, the correlation between the expression of target gene and related drugs IC50 was analyzed by Consortium for Classical Lutheran Education (CCLE) (http://www.ccle.org/) drug response database.

### Expression and network relationship between target genes and immune checkpoint genes

RNAseq data and corresponding clinical information for LUAD were obtained from TCGA dataset. *SIGLEC15*, *TIGIT, CD274*, *HAVCR2, PDCD1*, *CTLA4, LAG3* and *PDCD1LG2* are genes related to immune checkpoints. The expression values of these 8 genes were extracted to observe the expression of target genes related to immune checkpoints. According to the differential relationship between target genes and immune checkpoint genes, use the Gene Network Search function on the GenCLiP 3 website (http://ci.smu.edu.cn/genclip3/analysis.php) [35] to search for target genes and immune checkpoint genes with significant differences interaction networks and analyze possible regulatory relationships.

### Analysis of target gene and immune efficacy

The TCGA LUAD gene expression data were obtained, and the TIDE algorithm [36, 37] was used to predict the response of the high and low expression groups of the target gene to the predicted immune checkpoint inhibitor. TIDE uses a panel of gene expression signatures to assess 2 distinct tumor immune escape mechanisms, including tumor-infiltrating cytotoxic T lymphocyte (CTL) dysfunction and CTL rejection by immunosuppressive factors. High TIDE score, poor response to immune checkpoint blockade (ICB), and short survival after receiving ICB.

All the above statistical analysis and ggplot2 (v3.3.2) were completed using R program v4.0.3, *p* value < 0.05 was considered statistically significant.

## Results

### Identity of ImTRDG and functional enrichment results

**Fig. 2:**
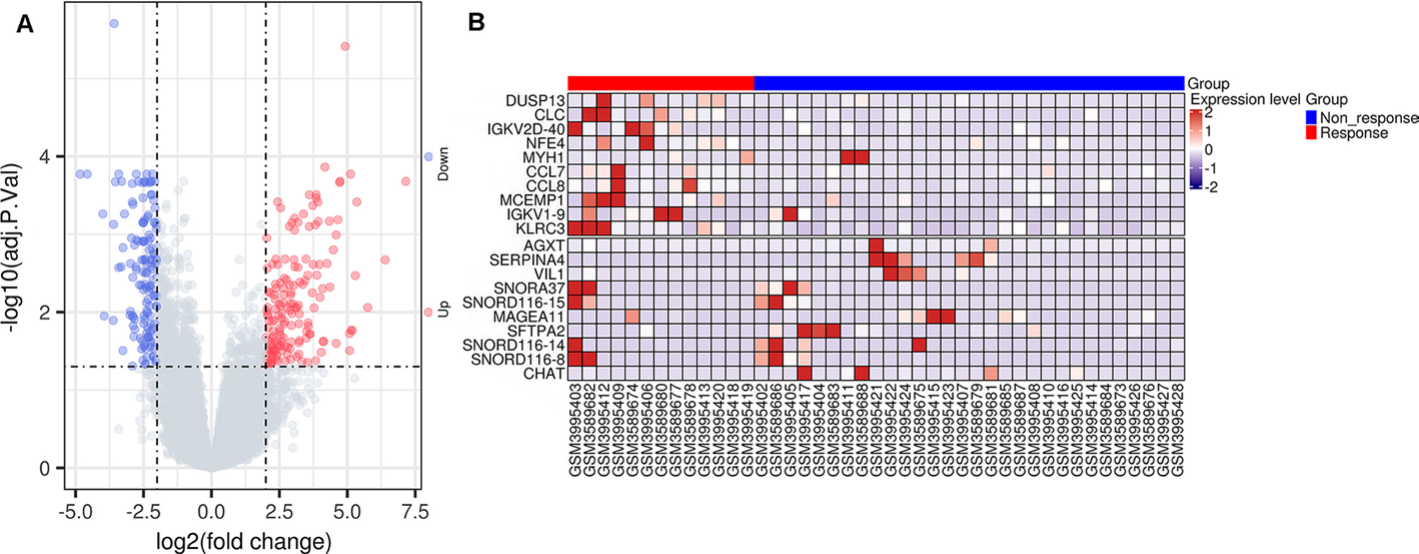
Visual presentation of imTRDG (**A**) Volcano plot of ImTRDG for *p* < 0.05, |LogFC > 2; (**B**) Heatmap of the top 20 ImTRDGs

The two datasets GSE135222 and GSE126044 in the CTR-DB database are about NSCLC patients who received immunochemotherapy, with a total of 43 patients. According to the effect of immunotherapy, they were divided into 13 responders (CR and PR) and 30 non-responders (SD and PD), as shown in Table 1. Through differential analysis and the set threshold, a total of 176 differential genes were screened, of which 72 were up-regulated genes and 104 genes were down-regulated. Figure 2A and B showed the volcano map and heat map of differential genes (Additional file 1: Supplementary table 1).

**Table 1:** Overview of receiving immunotherapy dataset information

Treatment response	Number	Data sets	Medication regimen	Pathological type
non-responder (SD and PD)	19	GEO:GSE135222	Immunotherapy	Lung non-small cell carcinoma
responder (CR and PR)	8	GEO:GSE135222	anti-PD-1/PD-L1	Lung non-small cell carcinoma
non-responder (SD and PD)	6	GEO:GSE126044	Nivolumab	Lung squamous cell carcinoma
non-responder (SD and PD)	5	GEO:GSE126044	Nivolumab	LUAD
responder (CR and PR)	3	GEO:GSE126044	Nivolumab	Lung squamous cell carcinoma
responder (CR and PR)	2	GEO:GSE126044	Nivolumab	LUAD

*CR* complete response, *PR* partial response, *SD* stable disease, *PD* progressive disease

**Fig. 3:**
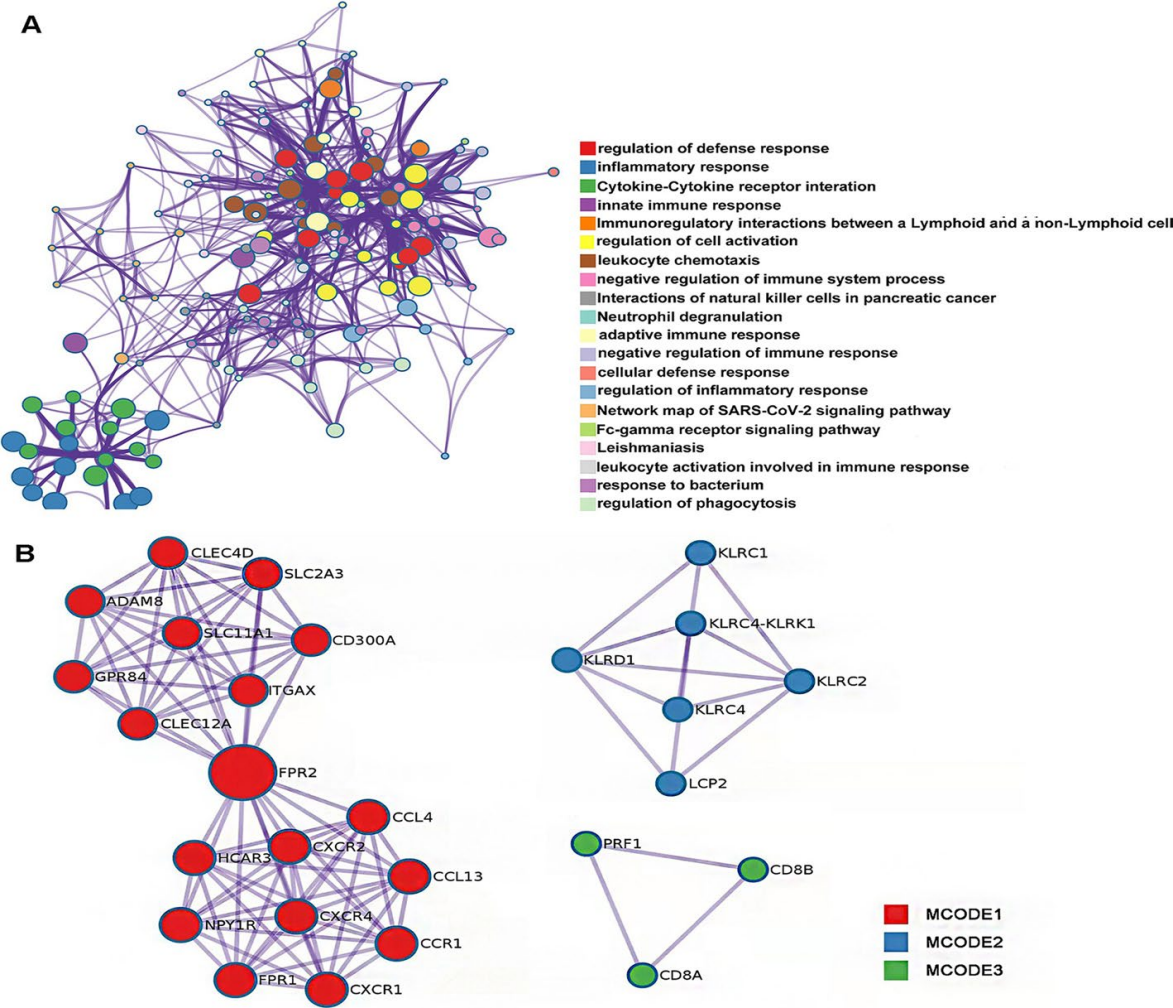
Visual presentation of imTRDG functional enrichment results. **A** Enriched Ontology Clusters Colored by Cluster type; **B** PPI MCODE components network

Through metascape enrichment analysis, the top 20 significant results were extracted, suggesting that most of the differential genes are involved in immune-related pathways (Fig. 3A, Additional file 2: Supplementary table 2), through the MOCODE algorithm, we obtained three densely connected PPI (protein–protein interaction) MCODE components, which are involved in neutrophil degranulation, regulation of natural killer cell mediated cytotoxicity and CD8 TCR (T cell receptor) pathway, respectively, as shown in Fig. 3B and Additional file 3: Supplementary table 3.

### Target genes for model screening and immune infiltration analysis

**Fig. 4:**
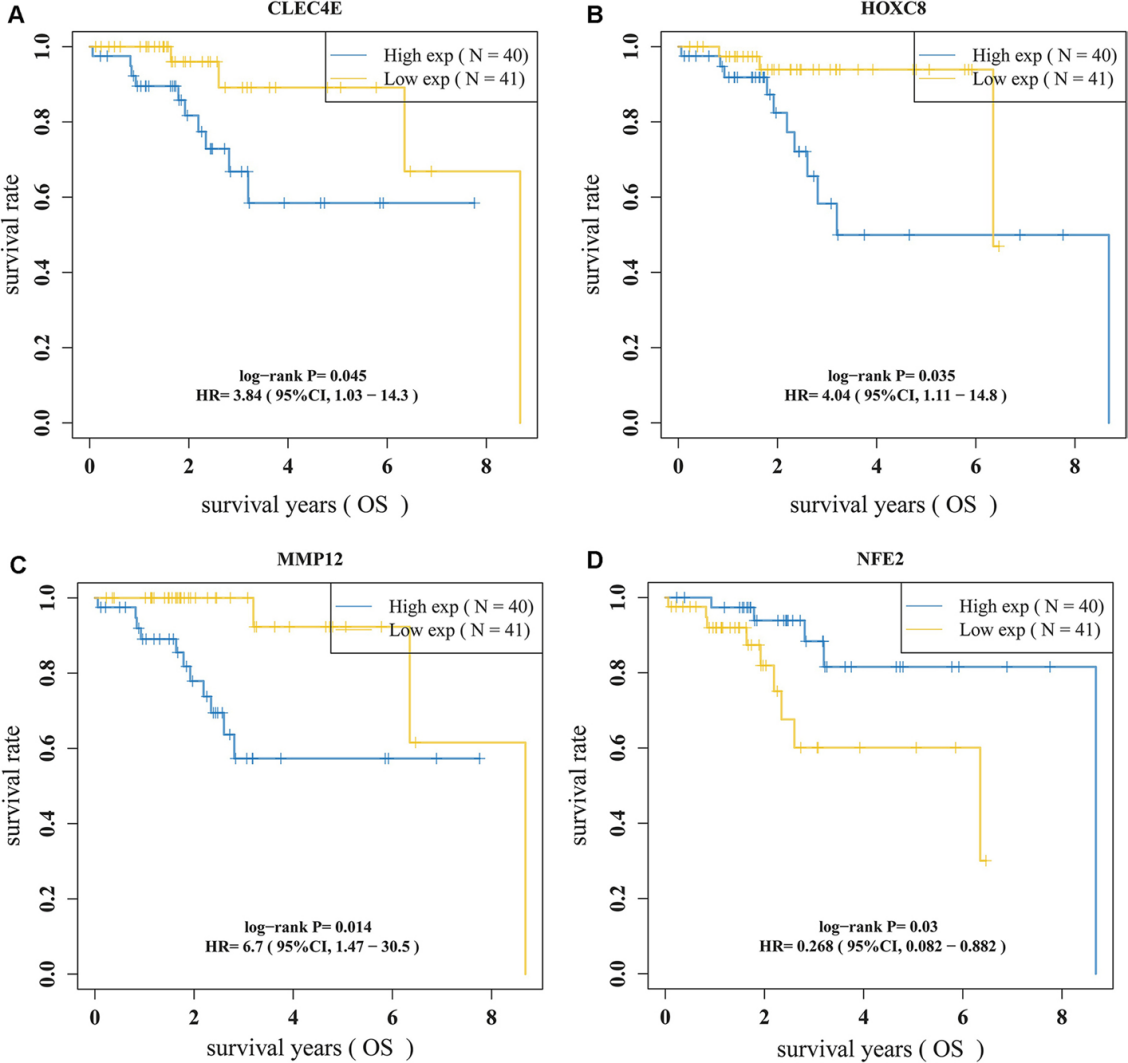
Univariate K-M survival curves of target genes. **A** K–M survival curves of *CLEC4E* in T1N0M0 LUAD; **B** K–M survival curves of HOXC8 in T1N0M0 LUAD; **C** K–M survival curves of MMP12 in T1N0M0 LUAD; **D** K–M survival curves of NFE2 in T1N0M0 LUAD

**Fig. 5:**
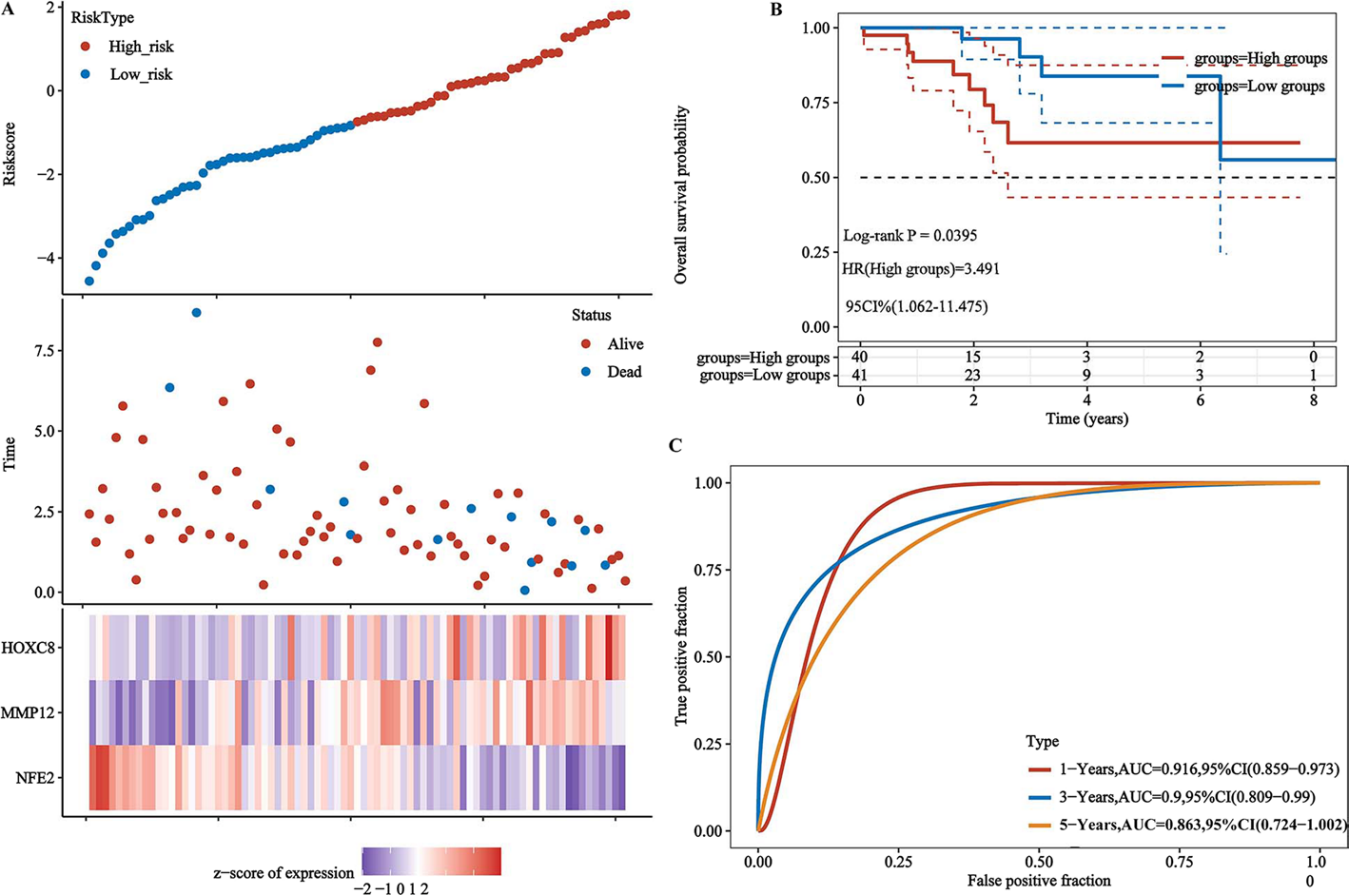
Multivariate K–M survival curves and ROC curves of risk model. **A** The survival time, survival status scatter plot and target gene distribution heat map corresponding to riskscore of different samples. The abscissa and ordinate represent genes, in which different colors represent the correlation coefficient, and the darker the color, the stronger the correlation; **B** Distribution of KM survival curves by prognostic risk model in T1N0M0 LUAD; **C** ROC curves and AUC values at different times in T1N0M0 LUAD risk model

**Fig. 6:**
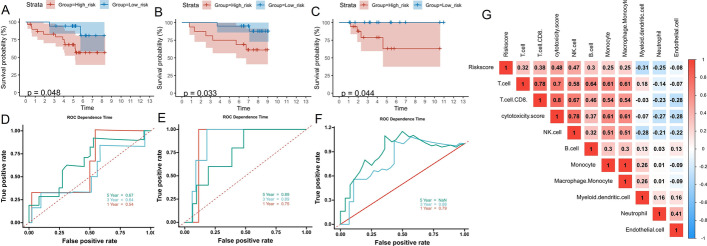
Multivariate K–M survival curves, ROC curves and immune infiltration landscapes of model genes in GSE50081, GSE11969 and GSE42127 validation sets. **A** Distribution of KM survival curves by prognostic risk model in GSE50081 T1N0M0 LUAD; **B** Distribution of KM survival curves by prognostic risk model in GSE11969 T1N0M0 LUAD; **C** Distribution of KM survival curves by prognostic risk model in GSE42127 T1N0M0 LUAD; **D** ROC curves and AUC values at different times in GSE50081 T1N0M0 LUAD; **E** ROC curves and AUC values at different times in GSE11969 T1N0M0 LUAD; **F** ROC curves and AUC values at different times in GSE42127 T1N0M0 LUAD; **G** The immune infiltration landscapes corresponding to riskscore in TCGA LUAD

After obtaining 176 ImTRDGs, univariate cox regression analysis was performed, and a total of 4 genes (*CLEC4E*, *HOXC8*, *MMP12* and *NFE2*) were obtained that were associated with the prognosis of T1N0M0 LUAD as shown in Fig. 4A–D, where the samples were divided into high expression group and low expression group according to the median value of gene expression, and P values of the univariate cox regression analysis (to obtain the prognostic gene set of *CLEC4E*, *HOXC8*, MMP12 and NFE2) was corrected by multiple hypothesis testing. Through multivariate cox and step functions, the risk model (Riskscore = −1.0068*NFE2 + 0.2741*MMP12 + 0.5986*HOXC8) constructed by 3 genes (*HOXC8*, *MMP12* and *NFE2*), 81 samples can be divided into high-risk and low-risk groups according to the median value of riskscore, survival analysis showed that the survival difference between the high-risk group and the low-risk group was statistically significant (HR = 3.491, 95%CI: 1.062–11.475, *P* = 0.0395). The 1-year, 3-year and 5-year ROC curve area was 0.916, 0.90 and 86.3, respectively (Fig. 5A–C). The GSE50081, GSE11969 and GSE42127data (Additional files 4, 5, 6: Supplementary tables 4, 5, 6) were used to verify the accuracy of the model. The results showed that the survival prognosis of patients in the high and low risk groups was statistically different (*p* = 0.048, *p* = 0.033 and *p* = 0.044, respectively) (Fig. 6A–C), and the 3-year ROC curve area was 0.64, 0.76 and 0.88, respectively (Fig. 6D–F), which was relatively stable. Combined with clinical data (age, gender and smoking status), univariate and multivariate cox regression analysis was performed, it has showed that *MMP12*, *NFE2* and *HOXC8* can be used as independent prognostic factors for T1N0M0 LUAD (Additional file 16: Fig. S1A, B). The correlation analysis between riskscore and immune infiltration scores showed that there was a good positive correlation between riskscore and cytotoxicity, NK.cell and CD8T cell scores (Fig. 6G, Additional file 7: Supplementary table 7).

### Differential expression and multi-type prognostic analysis

**Fig. 7:**
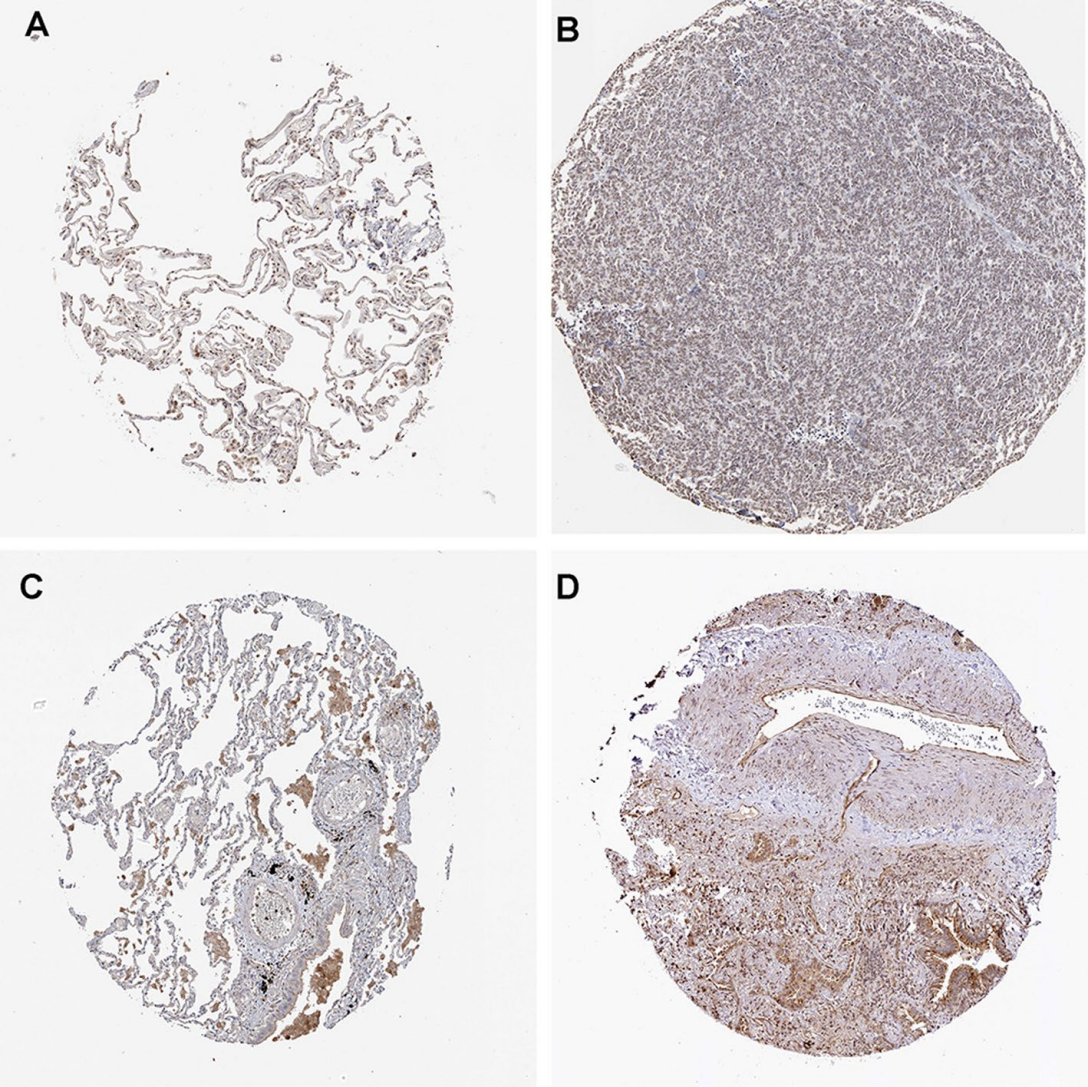
Immunohistochemical results of target genes proteins in normal lung and tumor tissues from the HPA database. **A** Immunohistochemical profile of NFE2 in normal lung; **B** Immunohistochemical profile of NFE2 in LUAD; **C** Immunohistochemical profile of HOXC8 in normal lung; **D** Immunohistochemical profile of HOXC8 in LUAD

**Fig. 8:**
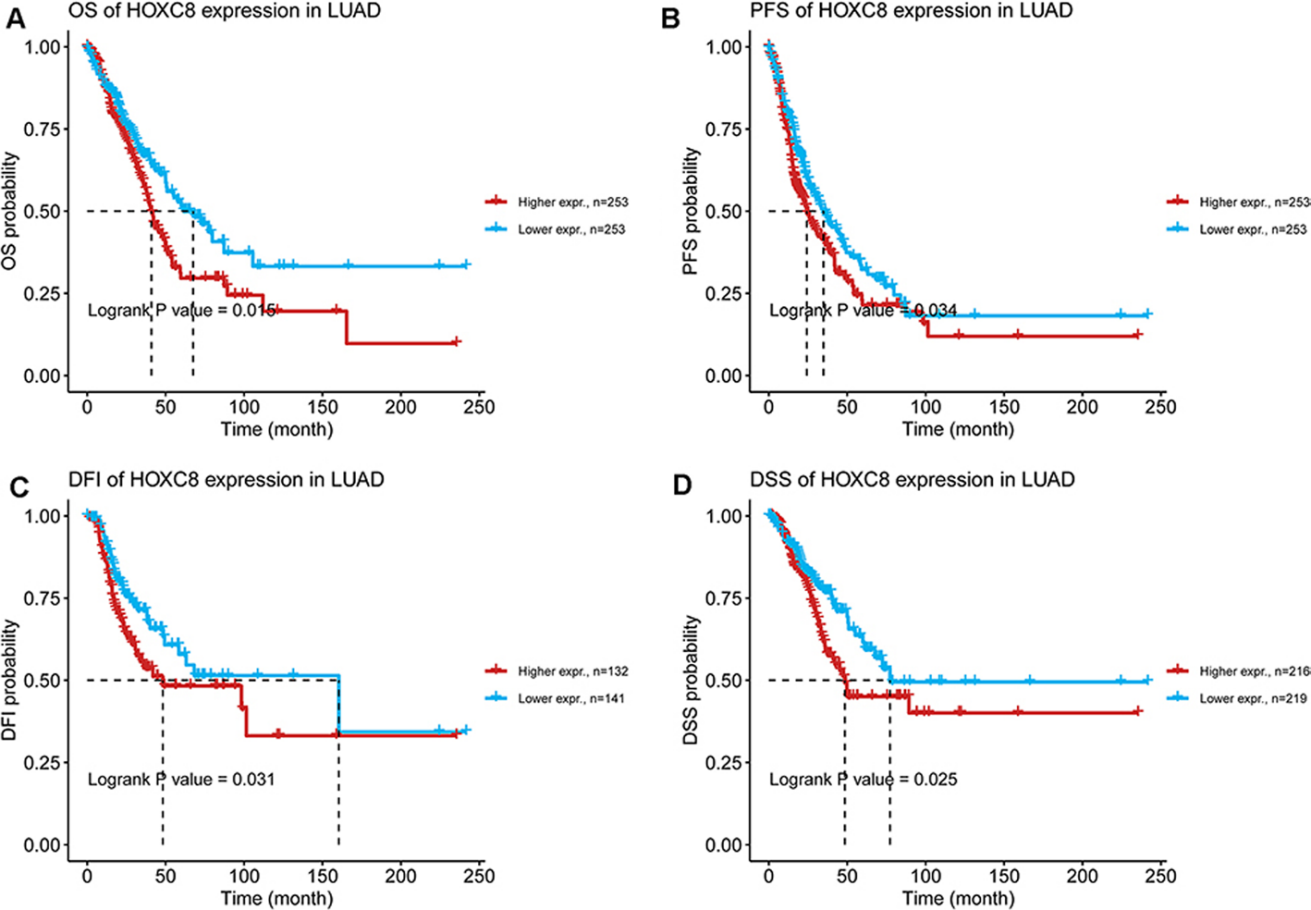
The multi-type K–M survival curves of target genes in LUAD. **A** OS K–M curves of HOXC8 in all stages of LUAD; **B** PFS K–M curves of HOXC8 in all stages of LUAD; **C** DFI K–M curves of HOXC8 in all stages of LUAD. **D** DSS K–M curves of HOXC8 in all stages of LUAD. *OS* overall survival, *PFS* progression free survival, *DSS* disease specific survival, *DFI* Disease Free Interval

The differential expression of the three genes in LUAD and normal samples from GTEx database was analyzed, and the results showed that *MMP12* and *HOXC8* were highly expressed in LUAD samples, while *NFE2* was low expressed in LUAD samples (Additional file 17: Fig. 2A, C). The immunohistochemical information of *NFE2* in LUAD and normal samples was searched by HPA, using HPA001914 antibody labeling, the results indicated that *NFE2* was moderately stained in normal alveolar tissue, but low in LUAD. Using HPA028911 antibody labeling, it was found that *HOXC8* was not stained in normal alveolar tissue, but moderately stained in alveolar macrophages, and *HOXC8* was moderately stained in LUAD tumor tissue. The results of immunohistochemistry and mRNA expression were consistent. (Fig. 7A–D) Survival analysis showed that *HOXC8* high expression was significantly correlated with poor OS, PFS, DSS, and DF of LUAD (Fig. 8A–D).

### Gene expression and pathway activity result

**Fig. 9:**
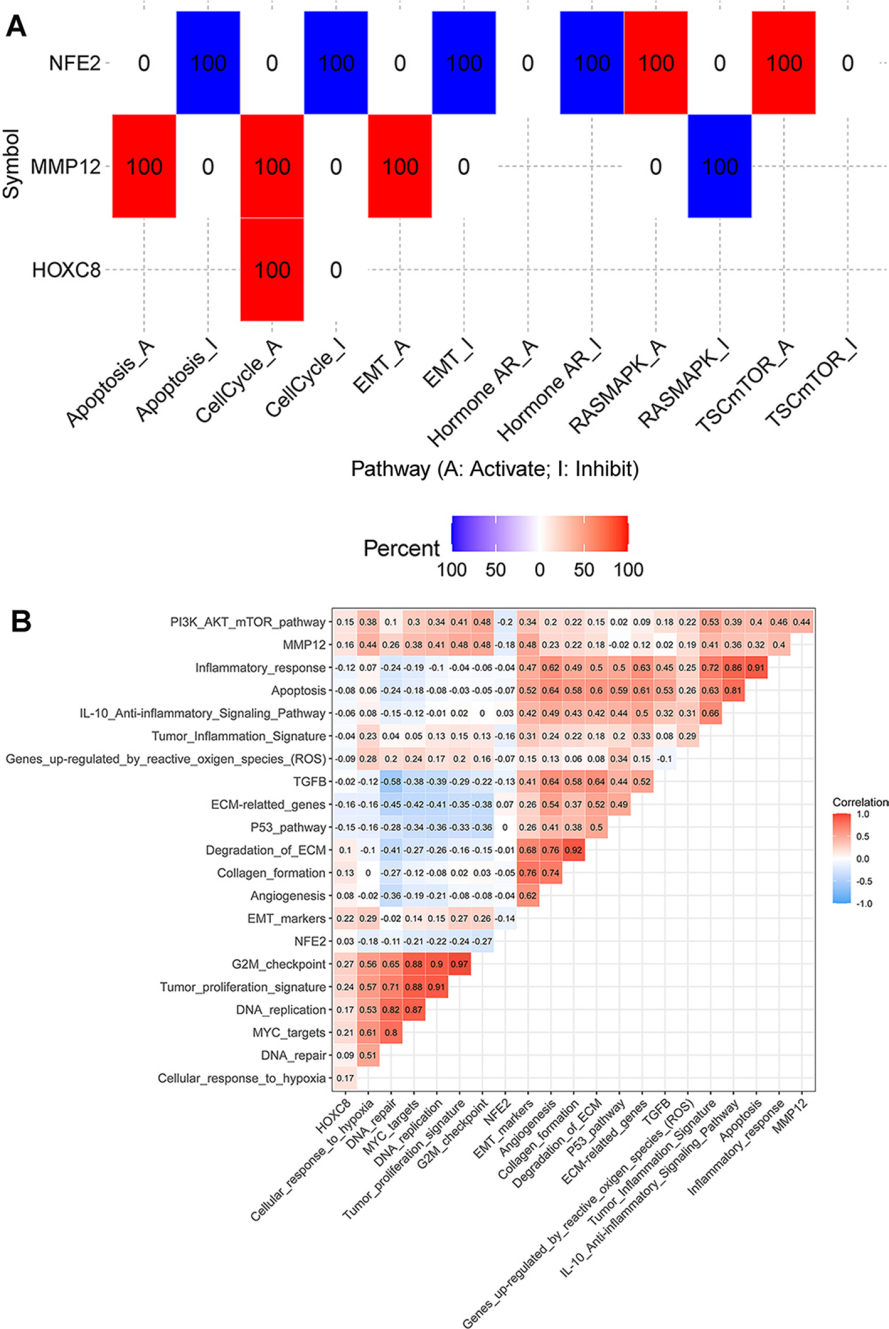
Heatmap of the correlation between target genes and immune infiltration. **A** Correlation heatmap of expression and pathway activity from GSCA. Percentage of cancer in which a gene have effect (FDR <  = 0.05) on the pathway in LUAD, the number in each cell indicates the percentage; **B** Correlation heatmap of gene expression and pathway activity from ssGSEA

GSCA–Expression and pathway activity module estimated difference of three genes expression between pathway activity groups (activation and inhibition). The results showed that *NFE2* may have inhibitory effects on the Apoptosis, CellCycle, *EMT* and Hormone *AR* pathways of LUAD, while it has an activation effect on the *MAPK* and *mTOR* pathways. *MMP12* has an activating effect on Apoptosis, CellCycle and *EMT* pathways of LUAD, and has an inhibitory effect on *MAPK* pathway, and *HOXC8* has an activating effect on CellCycle pathway (Fig. 9A, Additional file 8: Supplementary table 8). Through pathway ssGSEA analysis, the relationship between target genes and pathway scores was calculated and found that *MMP12* had positive correlation with Cellular_response_to_hypoxia, Tumor_proliferation_signature, G2M_checkpoint, Tumor_Inflammation_Signature and DNA_repair. NFE2 has negative correlation with Tumor_proliferation_signature and G2M_checkpoint, and *HOXC8* has positive correlation with Tumor_proliferation_signature and G2M_checkpoint (Fig. 9B, Additional file 9: Supplementary table 9).

### Copy Number Variation (CNV) and methylation survival prognostic analysis of target genes

**Fig. 10:**
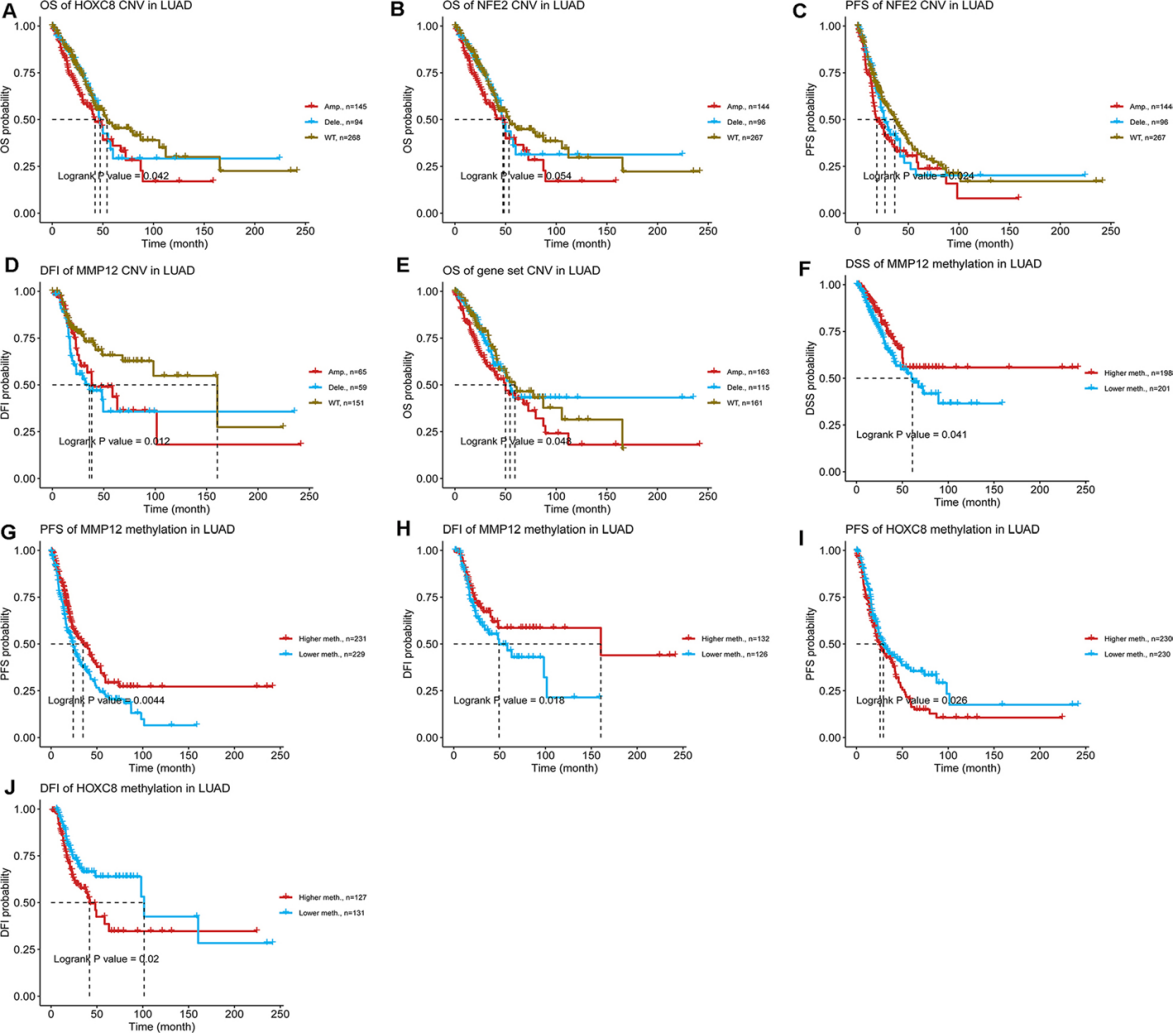
K–M survival prognosis curve of Copy Number Variation (CNV) and methylation of target genes. **A** OS of HOXC8 CNV in LUAD; **B** OS of NFE2 CNV in LUAD; **C** PFS of NFE2 CNV in LUAD; **D** DFI of MMP12 CNV in LUAD; **E** OS of gene set CNV in LUAD; **F** DSS of MMP12 methylation in LUAD; **G** PFS of MMP12 methylation in LUAD; **H** DFI of MMP12 methylation in LUAD; **I** PFS of HOXC8 methylation in LUAD; **J** DFI of HOXC8 methylation in LUAD. *OS* overall survival, *PFS* progression free survival, *DSS* disease specific survival, *DFI* Disease Free Interval

The summary of CNV of target genes in LUAD shown in the Table 2. The results of the CNV and LUAD survival prognostic analysis showed that compared with the WT group, the *HOXC8* and *NFE2* CNV groups were associated with poor OS (Fig. 10A, B), and *NFE2* CNV groups were also associated with poor PFS in LUAD (Fig. 10C). The *MMP12* CNV was associated with poor DFI (Fig. 10D). The detailed results are shown in Table 3; The results of CNV and survival prognostic analysis after the integration of the three genes showed that the gene set CNV was associated with poor OS in LUAD (Fig. 10E). Survival analysis showed that *MMP12* hypermethylation levels were associated with good DFS, DSS and DFI (Fig. 10F–H, Table4), and *HOXC8* hypermethylation levels were associated with poor PFS and DFI in LUAD (Fig. 10I, J, Table4).

**Table 2:** The summary of CNV of target genes in LUAD

Symbol	Total amp. (%)	Total dele. (%)	Hete amp. (%)	Hete dele. (%)	Homo amp. (%)	Homo dele. (%)
HOXC8	28.68217	18.21705	27.51938	18.02326	1.162791	0.193798
MMP12	26.16279	19.37985	24.22481	18.99225	1.937985	0.387597
NFE2	28.48837	18.60465	27.32558	18.41085	1.162791	0.193798

Total amp. (%): the percentage of samples with copy number amplification, including heterozygous and homozygous amplification; Total dele. (%): the percentage of samples with copy number deletion, including heterozygous and homozygous deletion;

Hete amp. (%): the percentage of samples with copy number heterozygous amplification; Hete dele. (%): the percentage of samples with copy number heterozygous deletion; Homo amp. (%): the percentage of samples with copy number homozygous amplification; Homo dele. (%): the percentage of samples with copy number homozygous deletion

**Table 3:** The detailed information of survival difference between CNV and wide type in LUAD

Symbol	sur_type	*p* value
HOXC8	OS	0.041727
HOXC8	PFS	0.019041
HOXC8	DSS	0.116383
HOXC8	DFI	0.564909
MMP12	OS	0.29116
MMP12	PFS	0.116586
MMP12	DSS	0.362409
MMP12	DFI	0.012319
NFE2	OS	0.05375
NFE2	PFS	0.024003
NFE2	DSS	0.134032
NFE2	DFI	0.651083

*OS* overall survival, *PFS* progression free survival, *DSS* disease specific survival, and *DFI* disease free interval

**Table 4:** The overall survival difference between higher and lower methylation groups in LUAD

Symbol	Tag	sur_type	*P*_value	HR	Group
HOXC8	cg19634247	OS	0.161562	1.247549	Higher meth
HOXC8	cg19634247	PFS	0.026401	1.341563	Higher meth
HOXC8	cg19634247	DSS	0.142748	1.341601	Higher meth
HOXC8	cg19634247	DFI	0.020919	1.700252	Higher meth
MMP12	cg20487452	OS	0.079336	0.755476	Lower meth
MMP12	cg20487452	PFS	0.004617	0.685559	Lower meth
MMP12	cg20487452	DSS	0.042944	0.662403	Lower meth
MMP12	cg20487452	DFI	0.019631	0.583485	Lower meth
NFE2	cg24762231	OS	0.538537	1.101948	Higher meth
NFE2	cg24762231	PFS	0.827304	0.971628	Lower meth
NFE2	cg24762231	DSS	0.743642	0.936438	Lower meth
NFE2	cg24762231	DFI	0.491236	0.854394	Lower meth

*OS* overall survival, *PFS* progression free survival, *DSS* disease specific survival, and *DFI* disease free interval, *meth.* Methylation

### Drug sensitivity analysis

**Fig. 11:**
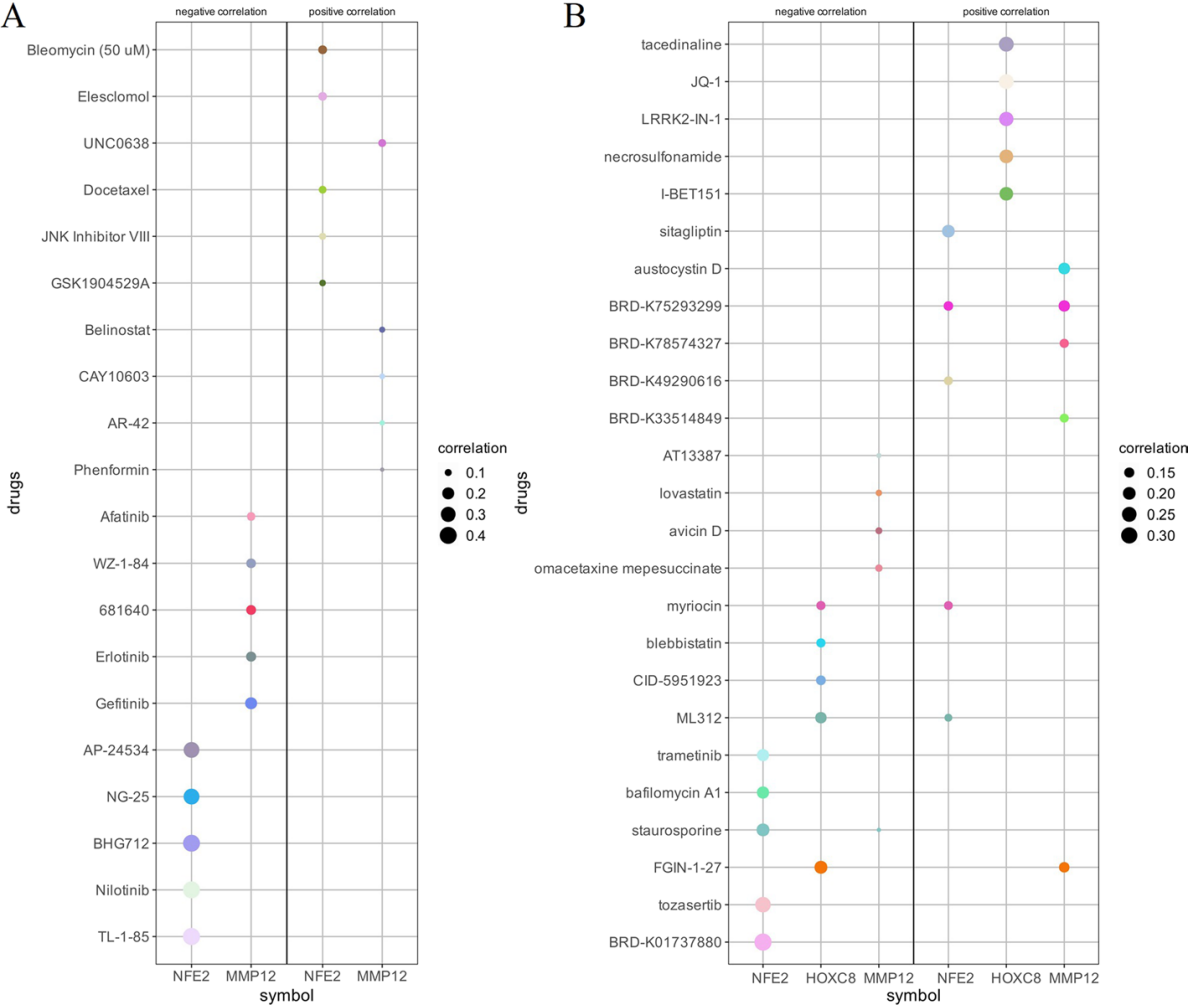
Distribution of IC50 of different drugs between high and low expression groups of target genes. **A** Distribution of IC50 of different drugs between high and low expression groups of target genes from GDSC; **B** Distribution of IC50 of different drugs between high and low expression groups of target genes from CTRP

From the GDSC database, we analyzed that *NFE2* mRNA expression was correlated with IC50 of Nilotinib, TL-1-85 and BHG712, and *MMP12* mRNA expression was negatively correlated with Gefitinib IC50 (Fig. 11A, Additional file 10: Supplementary table 10), however, no sensitive drugs related to HOXC8 were found; CTRP database analysis found that *NFE2* mRNA expression was negatively correlated with BRD-K01737880 IC50, *HOXC8* mRNA expression was positively correlated with tacedinaline, JQ-1 IC50 (Fig. 11B, Additional file 11: Supplementary table 11); CCLE database results indicated that the *FGFR* targeting drug TKI258 IC50 difference was statistically significant in the *HOXC8* mRNA high and low expression groups, In the MMP12 mRNA high and low expression groups, the IC50 differences of *c-MET* targeting drug PF2341066, *ALK* targeting drug TAE684 and *IGF1R* targeting drug AEW541 were statistically significant (Additional file 18: Figs. S3, S4, Additional files 12, 13: Supplementary tables 12, 13).

### Immune infiltration analysis

**Fig. 12:**
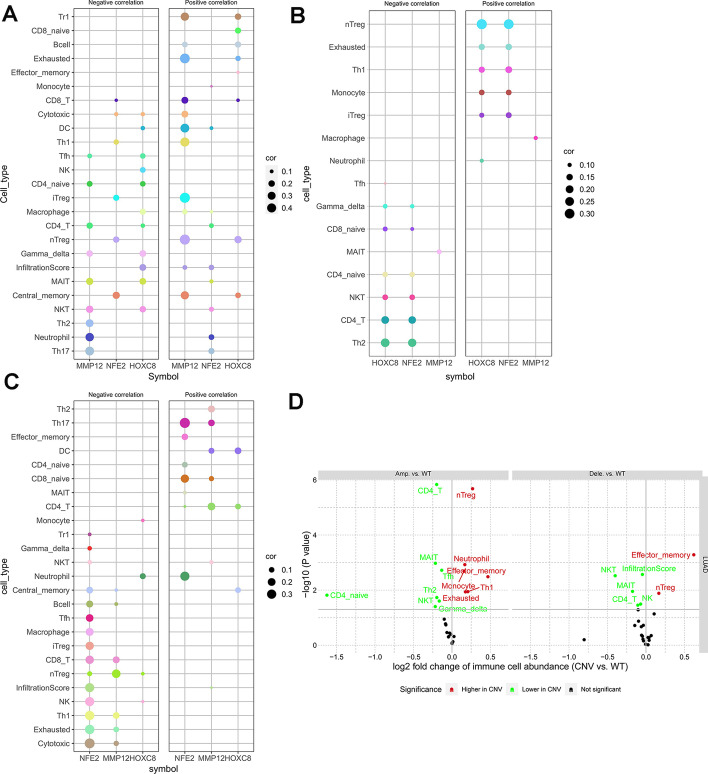
Bubble plot of the correlation between target genes and immune infiltration. **A** The correlation between target gene mRNA expression and immune infiltration; **B** The correlation between methylation level of target gene and immune infiltration; **C** The correlation between CNV status of target gene and immune infiltration; **D** The correlation of gene set CNV correlation with immune infiltration. The correlation P values in the above figures are all less than 0.05, which is statistically significant

Gene expression and immune infiltration analysis showed that *MMP12* was correlated with many immune infiltration components, among which it was positively correlated with nTreg, iTreg and Exhausted, and negatively correlated with Th17 and Th2. *NFE2* expression was negatively correlated with central_memory, *HOXC8* expression was positively correlated with nTreg, and negatively correlated with Gamma_delta and MAIT (Mucosal Associated Invariant T) (Fig. 12A, Additional file 14: Supplementary table 14).

The results of gene CNV and immune infiltration showed that *NFE2* and *HOXC8* CNV were positively correlated with nTreg and negatively correlated with *CD4*_T and Th2(Fig. 12B, Additional file 15: Supplementary table 15).

The results of gene methylation and immune infiltration analysis showed that *MMP12* methylation was negatively correlated with nTreg cells and positively correlated with *CD4* T cells, *NFE2* methylation was positively correlated with *Th17*, and negatively correlated with NK cells, Th1 cells, Cytotoxic and Exhausted cells, and *HOXC8* methylation was positively correlated with DCs. cells, *CD4* T cells were positively correlated (Fig. 12C, Additional file 16: Supplementary table 16).

After integrating the CNV results of the three genes, their relationship with immune infiltration was analyzed, and it was found that nTreg, exhausted, effector_memory, monocyte, neutrophil, Th1 cells aggregated in high CNV tumors, while *CD4* naive, Th2, Tfh, NKT (Natural killer T cell), Gamma_delta, NK, MAIT and *CD4* T aggregated in low CNV tumors (Fig. 12D).

### Expression relationship and network between target genes and immune checkpoint genes

**Fig. 13:**
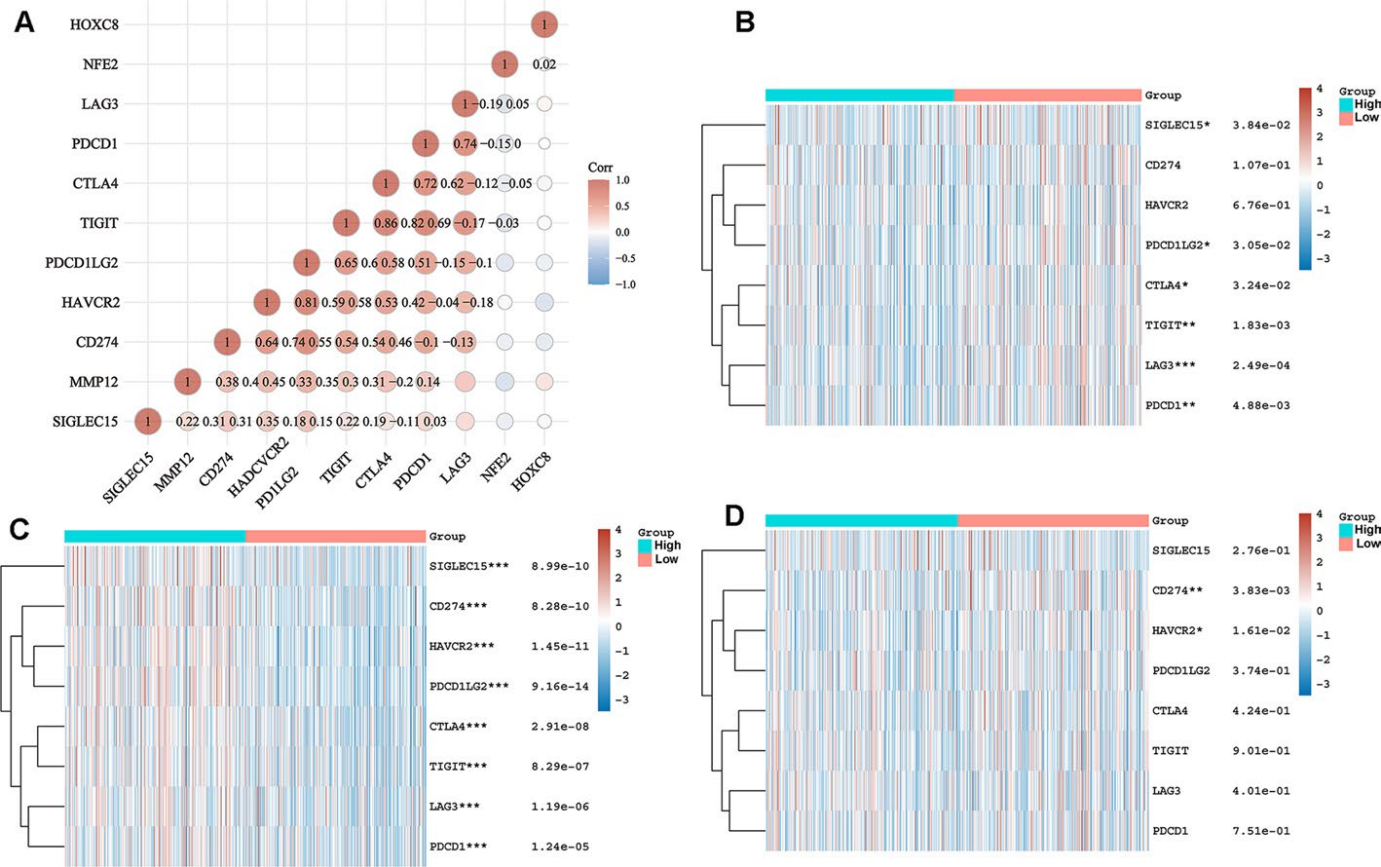
Heatmap of correlations between target genes and immune check-related genes. **A** Heatmap of linear correlations between target genes and immune check-related genes; **B** Heat map of differential expression of immune checkpoint-related genes between high and low HOXC8 expression groups; **C** Heat map of differential expression of immune checkpoint-related genes between high and low NFE2 expression groups; **D** Heat map of differential expression of immune checkpoint-related genes between high and low MMP12 expression groups. *p < 0.05, **p < 0.01, ***p < 0.001, asterisks (*) stand for significance levels

**Fig. 14:**
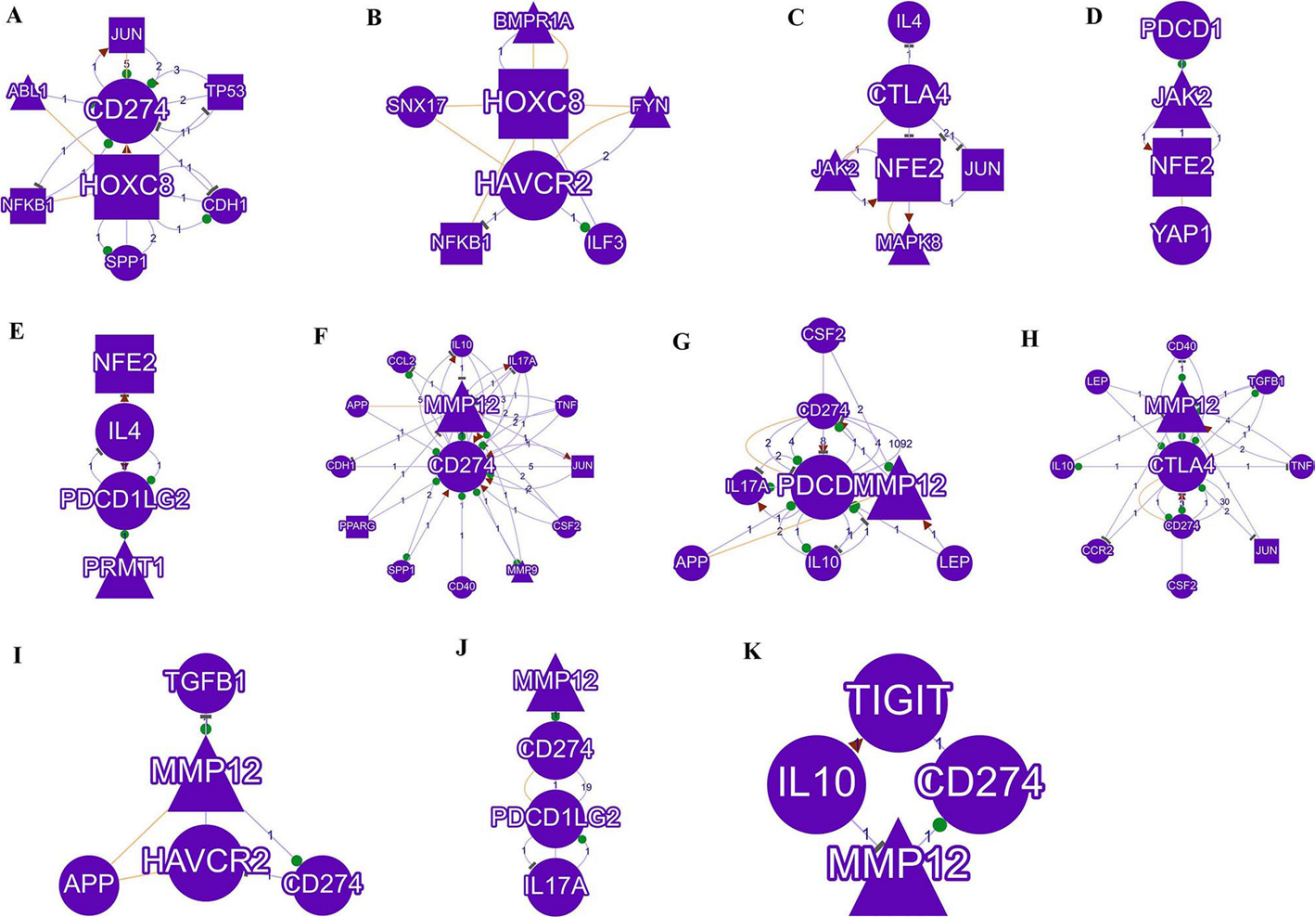
Regulatory networks of target genes and immune checkpoint-related genes. **A** Cross-talk between HOXC8 and CD274; **B** Cross-talk between HOXC8 and HAVCR2; **C** Cross-talk between NFE2 and CTLA4; **D** Cross-talk between NFE2 and PDCD1; **E** Cross-talk between NFE2 and PDCD1LG2; **F** Cross-talk between MMP12 and CD274; **G** Cross-talk between MMP12 and PDCD1; **H** Cross-talk between MMP12 and CTLA4; **I** Cross-talk between MMP12 and HAVCR2; **J** Cross-talk between MMP12 and PDCD1LG2; **K** Cross-talk between MMP12 and TIGIT. Circles represent genes, triangles represent enzymes, squares represent transcription factors, lines represent interactions, and the numbers on the lines represent the number of studies of interactions between genes that were experimentally validated or data-mined

The correlation analysis of the three genes and immune checkpoint genes found that only *MMP12* had a weak linear correlation with immune checkpoint genes (Fig. 13A). The target genes were divided into high and low expression groups according to their expression levels. Between the high and low expression groups of *HOXC8*, the expressions of *CD274* and *HAVCR2* were significantly different (Fig. 13B). The expressions of *HAVCR2*, *PDCD1LG*2, *CTLA4*, *TIGIT*, *LAG3* and *PDCD1* were all different in the *NFE2* high and low expression groups (Fig. 13C). In the high and low expression groups of *MMP12*, the expressions of *SIGLEC15*, *TIGIT*, *CD274*, *HAVCR2*, *PDCD1*, *CTLA4*, *LAG3* and *PDCD1LG2* were statistically different (Fig. 13D). Through the GenCLiP 3 website to analyze the potential regulatory networks of risk target genes and immune check genes, some of them have been confirmed by experiments, and some regulatory networks still need to be verified in the experimental area, as shown in the Fig. 14.

### Analysis of target gene and immune efficacy

**Fig. 15:**
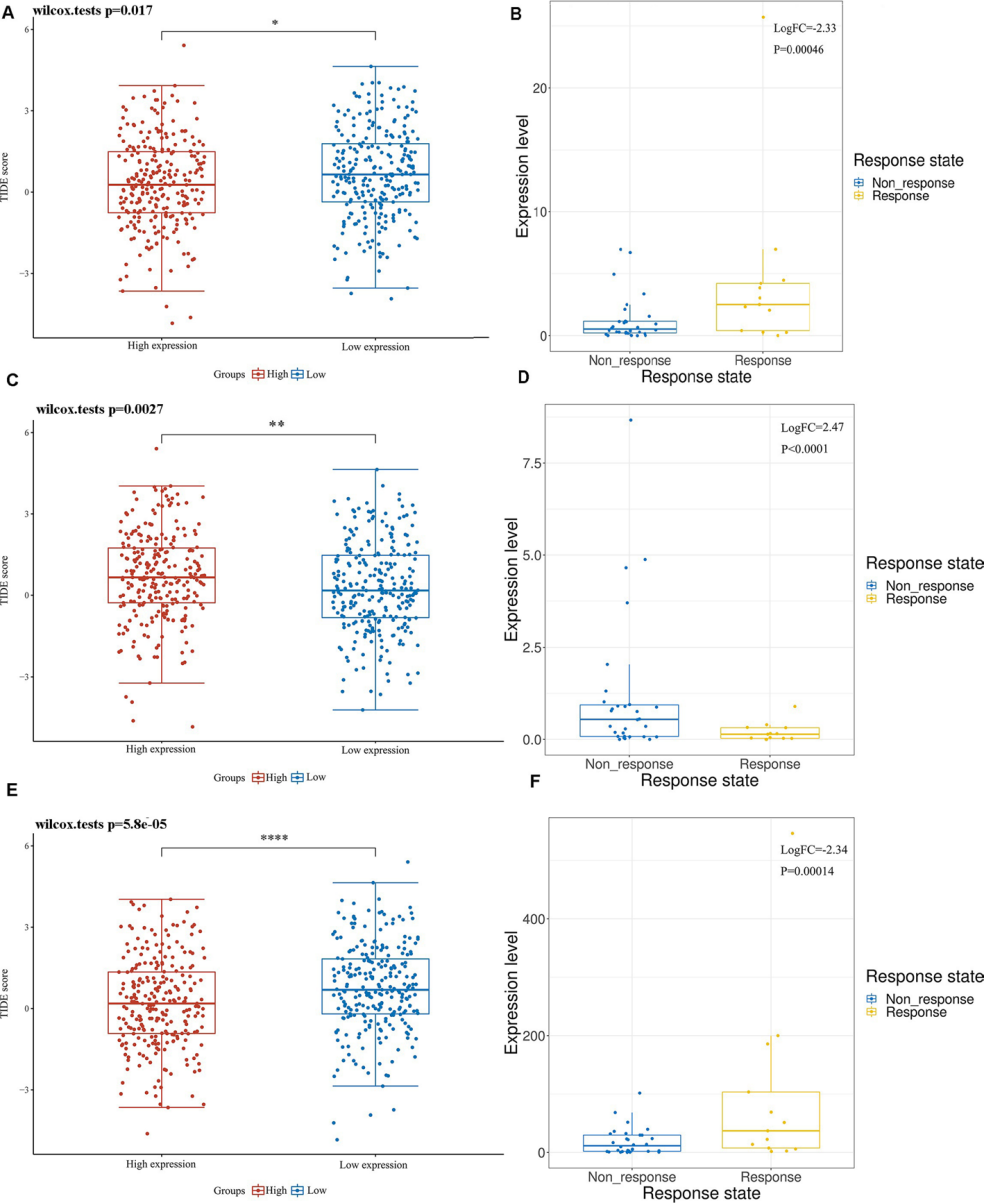
Prediction of the effect of immunotherapy under different expression of target genes. **A** Distribution of immune responses and immune scores in NFE2 high and low groups; **B** Distribution of NFE2 between immunotherapy responders and non-responders in the CTR-DB database; **C** Distribution of immune responses and immune scores in HOXC8 high and low groups; **D** Distribution of HOXC8 between immunotherapy responders and non-responders in the CTR-DB database; **E** Distribution of immune responses and immune scores in MMP12 high and low groups. **F** Distribution of MMP12 between immunotherapy responders and non-responders in the CTR-DB database. High TIDE score, poor response to immune checkpoint blockade (ICB), and short survival after ICB. *p < 0.05, **p < 0.01, ***p < 0.001, asterisks (*) stand for significance levels

The TIDE algorithm was used to calculate the response of the high and low expression LUAD of the three target genes to immunotherapy (Table 5). The results showed that 110 patients in the *NFE2* high expression group responded to immunotherapy, 147 patients did not respond to immunotherapy, 87 patients in the *NFE2* low expression group responded to immunotherapy, and 169 patients did not respond to immunotherapy. The TIDE score results showed that the TIDE score of the *NFE2* low expression group was higher, indicating that the effect of immunotherapy was poor, indicating that the high expression of *NFE2* may be a positive indicator of immunotherapy (Fig. 15A). This is consistent with the CTR-DB immunotherapy response differential gene results (Fig. 15B).

**Table 5:** Statistical table of immune responses of samples in different groups in prediction results

Response	NFE2		HOXC8		MMP12	
	high	low	high	low	high	low
True	110	87	85	112	119	78
False	147	169	172	144	137	179

In the *HOXC8* high expression group, 85 patients responded to immunotherapy, 172 patients did not respond to immunotherapy, 112 patients in the *HOXC8* low expression group responded to immunotherapy, and 144 patients did not respond to immunotherapy. The TIDE score results showed that the TIDE score was higher in the high *HOXC8* expression group, indicating that the immunotherapy effect was poor, which means that the high expression of *HOXC8* may be a negative indicator of immunotherapy (Fig. 15C), which is consistent with the CTR-DB immunotherapy response differential gene results (Fig. 15D).

In the *MMP12* high expression group, 119 patients responded to immunotherapy, 137 patients did not respond to immunotherapy, 78 patients in the *MMP12* low expression group responded to immunotherapy, and 179 patients did not respond to immunotherapy. The TIDE score results showed that the TIDE score of the *MMP12* low expression group was higher, indicating that the effect of immunotherapy was poor, indicating that the high expression of *MMP12* may be a positive indicator of immunotherapy (Fig. 15E). This is consistent with the CTR-DB immunotherapy response differential gene results (Fig. 15F).

## Discussion

With the advent of immunotherapy in recent years, the treatment and natural history of advanced NSCLC has been revolutionized, and immunotherapy for squamous cell carcinoma appears to yield better results than adenocarcinoma [38, 39]. In fact, in patients with driver-negative LUAD, the benefit of immune checkpoint inhibitors (ICIs) over previous standard chemotherapy has been demonstrated in first-line and further first-line therapy [40–42]. However, despite the overall benefit in survival outcomes, a large proportion of NSCLC patients were observed to experience disease progression. Exactly why this difference occurs and how to predict the effect of immunotherapy is still an important part of the ongoing research in the field of immunotherapy. Scientists have made great efforts to evaluate predictive biomarkers [43]. So far, only the high expression of programmed death ligand-1 demonstrated by immunohistochemistry has been confirmed for screening target populations even in different treatment stages and different immunotherapy regimens of LUAD predictive biomarkers. TMB (tumor mutational burden)/ bTMB (blood tumor mutational burden) has also been regarded as a predictor of immunotherapy. However, current studies have shown that TMB/bTMB as a predictor of ICIs treatment effect is still controversial. Exploratory analyses of CheckMate-026 [44] and POPLAR [45]/OAK [46] studies suggest that patients with high TMB/bTMB can benefit from immunotherapy, while the results of an exploratory analysis of the KEYNOTE series showed that TMB was not associated with efficacy, regardless of whether TMB was high or low, pembrolizumab plus chemotherapy in the first-line treatment of both squamous and non-squamous NSCLC patient survival benefit [47, 48]. However, Litchfield et al. collated all exome and transcriptome data of more than 1000 immunosuppressant treated patients in seven tumor types, and used standardized bioinformatics workflow and clinical results standards to verify multivariable predictors sensitive to immunotherapy. They found that clonal TMB was the strongest predictor of immunotherapy response, and they found that the expression of total TMB and *CXCL9* also had good predictive value, However, subclone TMB and somatic copy change load did not gain significant significance in pan cancer analysis, and these markers were internal determinants of tumors. Litchfield et al. also found new effective indicators in the tumor microenvironment. Through single cell sequencing of the tumor infiltrating lymphocytes of the clonal new antigen *CD8*, and transcriptional sequencing of bulk samples that are effective for immunotherapy, they finally determined that *CCR5* and *CXCL13* can be used as the internal immunotherapy sensitivity markers of T cells [49]. It has been reported that the clinical application of pembrolizumab in the treatment of advanced tumors was guided and the clinical efficacy of pembrolizumab was predicted based on the expression level of mismatch repair (MMR) [50]. The CheckMate-142 clinical study evaluated the efficacy of nivolumab monotherapy versus nivolumab in combination with ipilimumab in the treatment of metastatic colorectal cancer, in MSI-H colorectal cancer patients, ORR was better in both monotherapy and combination therapy groups than in patients with stable microsatellites [51]. Although MMR status may be used to predict the efficacy of immune checkpoint inhibitors, due to its low incidence in lung cancer, the predictive value of dMMR/MSI-H for lung cancer immunotherapy efficacy needs more research data to verify. In addition, some studies have explored the potential impact or possible correlation of new immune markers on immunotherapy. Some research shows that atezolizumab combined with bevacizumab and chemotherapy is an effective first line treatment in metadata NSCLC subgroups with mKRAS and cooccurrence *STK11* and/or *KEAP1* or *TP53* stations and/or high *PD-L1* expression [52]; There are also research findings that there were no associations between *SWI/SNF*(*ARID1A*, *PBRM1*) mut status and immunotherapy efficacy in the overall NSCLC cohort [53], and it has been reported that the clinical application of pembrolizumab in the treatment of advanced tumors was guided and the clinical efficacy of pembrolizumab was predicted based on the expression level of *MMR* [54]. Alterations of DNA damage response (*DDR*) pathways allow genomic instability, generate neoantigens, upregulate the expression of *PD-L1* and interact with signaling such as *STING* pathway, *ATM-ATR/CHK1* signaling, and the downstream component of *ATR/CHK1* signaling, signal transducer and activator of *STAT1/3*-interferon regulatory factor, is crucial for producing signal that can activate the generation of *PD-L1* mRNA at the transcriptional level [55].

The TME is composed of tumor cells, stromal cells (including vascular endothelial cells, pericytes, immune inflammatory cells, etc.) and extracellular matrix. The TME is not only the basis of tumor growth, invasion and metastasis [56], but also affects the clinical treatment effect of various cancers [57]. The tumor microenvironment has gradually become a research hotspot in recent years. Studies have shown that the interaction between cancer cells and the TME is bidirectional and dynamic, and the microenvironment has both promotion and inhibition on the occurrence and development of tumors. Like other malignant tumors, lung cancer is infiltrated with a large number of immune cells around the tumor, mainly T cells, macrophages and mast cells, while the relative content of plasma cells, natural killer cells and myeloid suppressor cells is relatively low [58, 59]. However, the specific cell composition has certain heterogeneity according to different tumor subtypes and patients [57]. The type, density, location and function of immune cells together constitute a specific immune context [60]. A large number of studies have shown that lymphocytes infiltrated by in situ tumors and metastases are closely related to tumor development and clinical outcomes of patients [12, 61]. The density of different cells in the immune microenvironment has a certain correlation with the survival of NSCLC, and has a strong prognostic value [57, 62].

We screened differential genes in response to immunotherapy, and functional enrichment analysis found that target genes are mainly involved in the process of immune stripping. Using TCGA data to build a prognostic model for early-stage LUAD, the model constructed from three genes has good predictive value. The immune infiltration of individual genes of interest can also be analyzed in all stages of LUAD. The predicted AUC values at 1, 3, and 5 years were 0.916 (95%CI 0.859–0.973), 0.9 (95%CI 0.809–0.99), and 0.863 (95%CI 0.724–1.002), respectively. Pathway activity analysis found that three genes were involved in EMT, tumor proliferation, cell cycle cycle, cell damage repair, *MAPK* and *mTOR* pathway to varying degrees.

*HOXC8* belongs to the HOX family, comprising 39 members in mammals, and the *HOXC8* protein is involved in many physiological and pathological processes, including embryogenesis and tumorigenesisv [63]. *HOXC8* has been reported to be dysregulated in various types of cancer, including breast, cervical, prostate, and ovarian cancer, and acts as a transcription factor to regulate the transcription of many genes [64]. *HOXC8* was significantly upregulated in NSCLC clinical specimens compared with normal tissues which is consistent with our TCGA database analysis results. And the upregulation of *HOXC8* played an important role in the tumorigenicity of NSCLC cell lines A549 and NCI-H460 [64]. Loss of E-cadherin expression is a hallmark of epithelial-mesenchymal transition (*EMT*) in tumor progression. Liu et al. [65] found that *HOXC8* could promote *EMT* in NSCLC, and E-cadherin was the target gene of *HOXC8*, the loss of E-cadherin promoted the growth and migration of NSCLC. The results of our pathway ssGSEA analysis also showed that *HOXC8* had a weak linear relationship with EMT pathway scores (Pearce correlation coefficient is 0.22, *p* < 0.05). Yu et al. [66]. found that *HOXC8* is a key biomarker for glioma diagnosis and prognosis through biological information, and the expression level of *HOXCs* is related to the infiltration of various immune cells. The prognostic value of *HOXC8* in glioma was further validated by qPCR and immunohistochemical data. The results of our immune infiltration analysis showed that *HOXC8* mRNA expression had a weak positive linear correlation with nTreg cell, and a weak negative linear correlation with Gamma_delta and MAIT cell. The results of gene CNV and immune infiltration showed that *HOXC8* CNV were positively correlated with nTreg and negatively correlated with *CD4* T and Th2. And the results of gene methylation and immune infiltration analysis showed that *HOXC8* was positively correlated with DCs. cells, *CD4* T cells. The correlation analysis between target genes and immune checkpoints showed that the expressions of *CD274* and *HAVCR2* were significantly different between high and low expression groups of *HOXC8*. TIDE analysis suggested that *HOXC8* may be a negative indicator of immunotherapy, which was basically consistent with the results of immune infiltration analysis. Although there is no strong linear relationship between *HOXC8* and immune checkpoint-related genes, the GenCLiP 3 website analysis found that *HOXC8* may have a complex regulatory network with immune checkpoint-related genes. In addition, drug sensitivity analysis found that *HOXC8* may affect the antitumor effect of multiple drugs. Experiments related to *HOXC8* methylation, CNV and immune infiltration of LUAD are still blank, and further basic experiments need to be carried out to prove it.

*NFE2* is a Protein Coding gene. Diseases associated with *NFE2* include Erythroleukemia and Polycythemia. Among its related pathways are Response to elevated platelet cytosolic Ca2+ and Hematopoietic Stem Cell Differentiation [67, 68]. There are few reports on the relationship between *NFE2* and tumors. Wang et al. [69]. analyzed lung cancer transcriptome sequencing and genomic data and found a novel *R3HDM2-NFE2* fusion in the H1792 lung cancer cell line. Lung tissue microarray revealed that 2 of 76 lung cancer patients had genomic rearrangements at the *NFE2* locus, and when *NFE2* was knocked down, it reduced the proliferation and invasion of H1792 cells. Dou et al. [70]. found that *NFE2* members bind to the antioxidant response element region and activate the expression of target genes. Through bioinformatics analysis, they showed that *NFE2* members mainly focus on transcriptional coactivator activities. The mRNA expression of *NFE2* members was significantly correlated with the immune infiltration of *CD4*+ T cells, *CD8*+ T cells, B cells, macrophages and neutrophils in Ovarian Cancer. The results of our immune infiltration analysis showed that *NFE2* expression was negatively correlated with Central_memory. Central memory T cells which are restricted to the secondary lymphoid tissues and blood are with long-term memory generated after naive T cells are activated by antigens, and can home to lymph nodes to receive antigen re-stimulation. Continue to generate large numbers of alloantigen-bearing clonal effector memory T cells upon restimulation. In 2005, Klebanoff CA et al. first proved that Central memory T cells have super anti-tumor ability [71]. In 2012, clinical studies such as the National Institutes of Health (NIH) found that Central memory T cells and their derived clonal T cells are highly effective anti-tumor cells. Tumor immune T cells [72]. Collecting the results of our analysis, we hypothesized that *NFE2* may be associated with tumor tertiary lymph nodes and circulating tumor cells in LUAD cells. The results of gene CNV and immune infiltration showed that *NFE2* CNV were positively correlated with nTreg and negatively correlated with *CD4*_T and Th2. And the results of gene methylation and immune infiltration analysis showed that NFE2 was positively correlated with Th17, and negatively correlated with NK cells, Th1 cells, Cytotoxic and Exhausted cells. The correlation analysis between target genes and immune checkpoints showed that the expressions of *HAVCR2*, *PDCD1LG2*, *CTLA4*, *TIGIT*, *LAG3* and *PDCD1* were all different in the *NFE2* high and low expression groups. TIDE analysis suggested that *NFE2* may be a positive indicator of immunotherapy, which was basically consistent with the results of immune infiltration analysis. The above dry analysis results still need experiments to enhance convincing.

Matrix metalloproteinases (*MMPs*) are a group of more than 20 proteolytic enzymes that degrade the extracellular matrix and facilitate invasion through the basement membrane [73, 74]. This ability of *MMPs* to remodel the extracellular milieu has led to extensive studies of their role in carcinogenesis. In NSCLC, *MMPs* are implicated in tumor invasion and metastasis through their ability to remodel and degrade the extracellular matrix and mediate cell–cell adhesion [75, 76]. In addition to disrupting the basement membrane, *MMPs* have been shown to influence the microenvironment of cells through complex cell–cell and cell–matrix interactions, by altering cell signaling and regulating cytokines, growth factors, and angiogenic factors [77]. Hofmann et al. [78] found that *MMP12* expression was significantly increased in tumors compared with corresponding lung tissues, and *MMP12* expression was significantly associated with local recurrence and metastatic disease. Multivariate Cox regression analysis showed that *MMP12* expression was an independent prognostic factor for tumor recurrence-free interval. Immunohistology identified *MMP12* protein in NSCLC only in tumor cells. Hung et al. [79]. found that nontoxic concentrations of penfluidol reduced LUAD cell migration, invasion, and adhesion. A protease array screen identifies *MMP12* as a potential target of penfluridor to modulate LUAD cell motility and adhesion. Mechanistic studies showed that penfluridol downregulates *MMP12* expression by inhibiting the urokinase plasminogen activator (uPA)/uPA receptor/transforming growth factor-beta/Akt axis, thereby reversing *MMP12*-induced EMT. Subsequent analysis of clinical LUAD samples revealed a positive correlation between *MMP12* and mesenchymal-related gene expression levels. In addition, some studies have found that *MMP12* may be involved in the *MAPK* pathway to affect cell damage and repair [80, 81]. These findings are consistent with our pathway activity analysis results. Regulatory T cells (Tregs) are a subset of immune cells, including nTregs and iTregs, both of which play a role in suppressing immunity and promote tumor progression by suppressing antitumor immune responses [82]. Kim et al. [83] used an anti-ST2 antibody to deplete Tregs in mouse lung tumors and found that local Tregs depletion resulted in a significant reduction in lung tumor burden. Immune responses following depletion of Tregs in tumors showed restoration of NK cell activity, enhanced Th1 activity, increased *CD8* cytotoxic T cell responses, and decreased expression of Mmp12. Our immune infiltration analysis found that *MMP12* showed a positive linear relationship with nTreg and iTreg, indicating that high expression of *MMP12* may mean increased nTreg and iTreg, promoting tumor growth, suggesting that *MMP12* may be a negative factor for immunotherapy, and our TIDE The analysis found that the higher the expression of *MMP12*, the higher the TIDE score and the worse the immunotherapy effect, which is consistent with the above findings. These data suggest that therapeutic strategies targeting activated Tregs in lung cancer have the potential to inhibit tumor progression by enhancing antitumor immunity. In addition, we analyzed the relationship between *MMP12* methylation levels and immune infiltration and found that MMP12 methylation was negatively correlated with nTreg cells and positively correlated with *CD4* T cells.The correlation analysis between target genes and immune checkpoints showed that the expressions of *SIGLEC15*, *TIGIT*, *CD274*, *HAVCR2*, *PDCD1*, *CTLA4*, *LAG3* and *PDCD1LG2* were significantly different between high and low expression groups of *MMP12*. The GenCLiP 3 website analysis found that *MMP12* may have a complex regulatory network with immune checkpoint-related genes. In addition, drug sensitivity analysis found that *MMP12* may affect the antitumor effect of multiple drugs. However, the above analysis results still need accurate experimental data to verify.

## Conclusions

In conclusion, our bioinformatic results suggest that the early-stage LUAD prognostic model constructed by *MMP12*, *NFE2*, and *HOXC8* has good predictive value; *MMP12*, *NFE2*, and *HOXC8* are involved in the formation and growth pathway of LUAD to varying degrees and may affect the The effect of some antitumor drugs; the mRNA expression, methylation level and CNV status of *MMP12*, *NFE2*, H*OXC8* have a certain linear relationship with some immune infiltration components, which may be involved in the immune regulation of tumors; *MMP12*, *NFE2*, *HOXC8* and immune examination Dot-related genes have complex regulatory networks that affect immunotherapy and are expected to be markers of immunotherapy, which are worthy of further experimental research. Inevitably, there are some limitations in this study. First of all, we use bioinformatics methods to preliminarily explore the immune regulatory functions that the three target genes may participate in and predict the effect of NSCLC immunotherapy, the bioinformatics analysis still lacks strong convincing power and needs to be verified by subsequent experiments. At the same time, since the initially included immunotherapy samples have no long-term survival data, it is impossible to prove the predictive value of the target gene on the long-term survival of NSCLC immunotherapy. In addition, during the construction of T1N0M0 LUAD prognosis model, the number of eligible samples included was limited, which may have some analysis bias. Although three data sets were used for verification, and good prediction results were obtained, the evidence of survival data with large sample size is still needed.

## Supplementary Information

**Additional file 1**.** Supplementary table 1**. Immunotherapy response differential genes.

**Additional file 2**. **Supplementary table 2**. Metascape enrichment analysis.

**Additional file 3**. **Supplementary table 3**. PPI MCODE components.

**Additional file 4**.** Supplementary table 4**. The gene expression survival and rickscore results of GSE50081.

**Additional file 5**.** Supplementary table 5**. The gene expression survival and rickscore results of GSE11969.

**Additional file 6**.** Supplementary table 6**. The gene expression survival and rickscore results of GSE42127.

**Additional file 7**. **Supplementary table 7**. The correlation between immune infiltration and rickscore.

**Additional file 8**.** Supplementary table 8**. pathway ssGSEA score.

**Additional file 9**. **Supplementary table 9**. Gene expression and pathway activity result from GSCA.

**Additional file 10**.** Supplementary table 10**. CTRP drug IC50 and expression table.

**Additional file 11**. **Supplementary table 11**. GDSC drug IC50 and expression table.

**Additional file 12**.** Supplementary table 12**. CCLE IC50 and MMP12 exprression table.

**Additional file 13**.** Supplementary table 13**. CCLE IC50 and HOXC8 exprression table.

**Additional file 14**.** Supplementary table 14**. Correlation between immunity and mRNA of target genes.

**Additional file 15**.** Supplementary table 15**. Correlation between immunity and CNV of target genes.

**Additional file 16**.** Supplementary table 16**. Correlation between immunity and methylation of target genes.

**Additional file 17**. The figure of univariate and multivariate cox analysis of target genes expression and clinical characteristics. **Figure S1**. Univariate and multivariate cox analysis of target genes expression and clinical characteristics. A Univariate cox analysis of gene expression and clinical characteristics; B. multivariate cox analysis of gene expression and clinical characteristics.

**Additional file 18**.The figurge of differential expression distribution of target genes in all stages of tumors and adjacent tissues from HPA database. **Figure S2**. Differential expression distribution of target genes in all stages of tumors and adjacent tissues. A. Differential expression of MMP12; B. Differential expression of HOXC8; C. Differential expression of NFE2.

**Additional file 19**.The drug sensitivity analysis of HOXC8 from CCLE database. **Figure S3**. Correlation bubble chart for drug sensitivity analysis of IC50 of different drugs in HOXC8 high and low groups from CCLE database.*p < 0.05, **p < 0.01, ***p < 0.001, asterisks (*) stand for significance levels.

**Additional file 20**. The drug sensitivity analysis of MMP12 from CCLE database. **Figure S4.** Correlation bubble chart for drug sensitivity analysis of IC50 of different drugs in MMP12 high and low groups from CCLE database. *p < 0.05, **p < 0.01, ***p < 0.001, asterisks (*) stand for significance levels.

## Data Availability

The datasets (GSE135222, GSE126044, GSE50081, GSE11969 and GSE42127) generated and analysed during the current study are available in the GEO dataset repository. https://www.ncbi.nlm.nih.gov/gds. The original contributions presented in the study are included in the article/Additional files 1, 2, 3, 4, 5, 6, 7, 8, 9, 10, 11, 12, 13, 14, 15, 16, 17, 18, 19, 20. Further inquiries can be directed to the corresponding authors. The Additional files 1, 2, 3, 4, 5, 6, 7, 8, 9, 10, 11, 12, 13, 14, 15, 16, 17, 18, 19, 20 for this article can be found online.

## Abbreviations

CTR-DB: Cancer Treatment Response Gene Signature DataBase
LUAD: Lung Adenocarcinoma
TCGA: The Cancer Genome Atlas
MOCODE: Molecular Complex Detection
*MMP12*: Matrix Metallopeptidase 12
*NFE2*: Nuclear Factor, Erythroid 2
*HOXC8*: Homeobox C8
ROC: Receiver Operating Characteristic
GEO: The Gene Expression Omnibus
CNV: The Copy Number Variation
TME: Tumor Microenvironment
TIDE: Tumor Immune Dysfunction and Exclusion
NSCLC: Non-small Cell Lung Cancer
FDA: The Food and Drug Administration
ICIs: Immune Checkpoint Inhibitors
*PD-L1*: Programmed cell death ligand 1
*P**D-1*: Programmed cell death 1
ImTRDG: Immunotherapy Response Differential Genes
CR: Complete Rresponse
PR: Partial Response
SD: Stable Disease
PD: Progressive Disease
HR: Hazard Ratios
CI: Confidence Intervals
FDR: False Discovery Rate
HPA: The Human Protein Atlas
OS: Overall Survival
PFS: Progression Free Survival
DSS: Disease Specific Survival
DFI: Disease Free Interval
RPPA: Reverse Phase Protein Array
GSCA: The Gene Set Cancer Analysis
PAS: Pathway Activity Score
SsGSEA: Single Aample Gene Set Enrichment Analys
TSC: Tuberous Sclerosis
*MTOR*: Mammalian Target of Rapamycin
*RTK*: Receptor Tyrosine Kinases
*MAPK*: Mitogen-Activated Protein Kinase
*PI3K*: Phosphoinositide-3 Kinase
*ER*: Estrogen Receptor
*AR*: Androgen Receptor
*EMT*: Epithelial–Mesenchymal Transition
DNA: DeoxyriboNucleic Acid
mRNA: Messenger Ribonucleic Acid
CTRP: Genomics of Therapeutics Response Portal
GDSC: Genomics of Drug Sensitivity in Cancer
*SIGLEC15*: Sialic Acid Binding lg Like Lectin 15
*TIGIT*: T cell Immunoreceptor with Ig and ITIM domains
*HAVCR2*: Hepatitis A virus Cellular Receptor 2
*PDCD1*: Programmed cell death 1
*CTLA4*: Cytotoxic T-lymphocyte Associated Protein 4
LA*G3*: Lymphocyte activating 3
*PDCD1LG2*: Programmed cell death 1 ligand 2
CTL: Cytotoxic T Lymphocyte
ICB: Immune Checkpoint Blockade
PPI: Protein–Protein Interaction
*CLEC4E*: C-type Lectin Domain family 4 member E
GTEx: Genotype-Tissue Expression
*ALK*: Anaplastic Lymphoma Kinase
*FGFR*: Fibroblast Growth Factor Receptor
CCLE: Cancer Cell Line Encyclopedia
MAIT: Mucosal Associated Invariant T
NKT: Natural Killer T cell
TMB: Tumor Mutational Burden
bTMB: Blood Tumor Mutational Burden
MMR: Mismatch Repair
*R3HDM2*: R3H domain Containing 2
*MMPs*: Matrix Metalloproteinases
*CXCL9*: C-X-C motif chemokine ligand 9
*CCR5*: C–C motif chemokine receptor 5
*CXCL13*: C-X-C motif chemokine ligand 13
STK11: Serine/threonine kinase 11
KEAP1: Kelch like ECH associated protein 1
ARID1A: AT-rich interaction domain 1A
PBRM1: Polybromo 1
STING1: Stimulator of interferon response cGAMP interactor 1
CHK1: Checkpoint kinase 1
ATR: ATR serine/threonine kinase
ATM: ATM serine/threonine kinase
STAT1: Signal transducer and activator of transcription 1
STAT3: Signal transducer and activator of transcription 3
DDR: DNA damage response
