# Supplementary figures and images for: Genomic and immunogenomic analysis of three prognostic signature genes in LUAD
Source: BMC Bioinformatics. 2023 Jan 17;24:19. doi: 10.1186/s12859-023-05137-y (PMC9843910; doi:10.1186/s12859-023-05137-y)

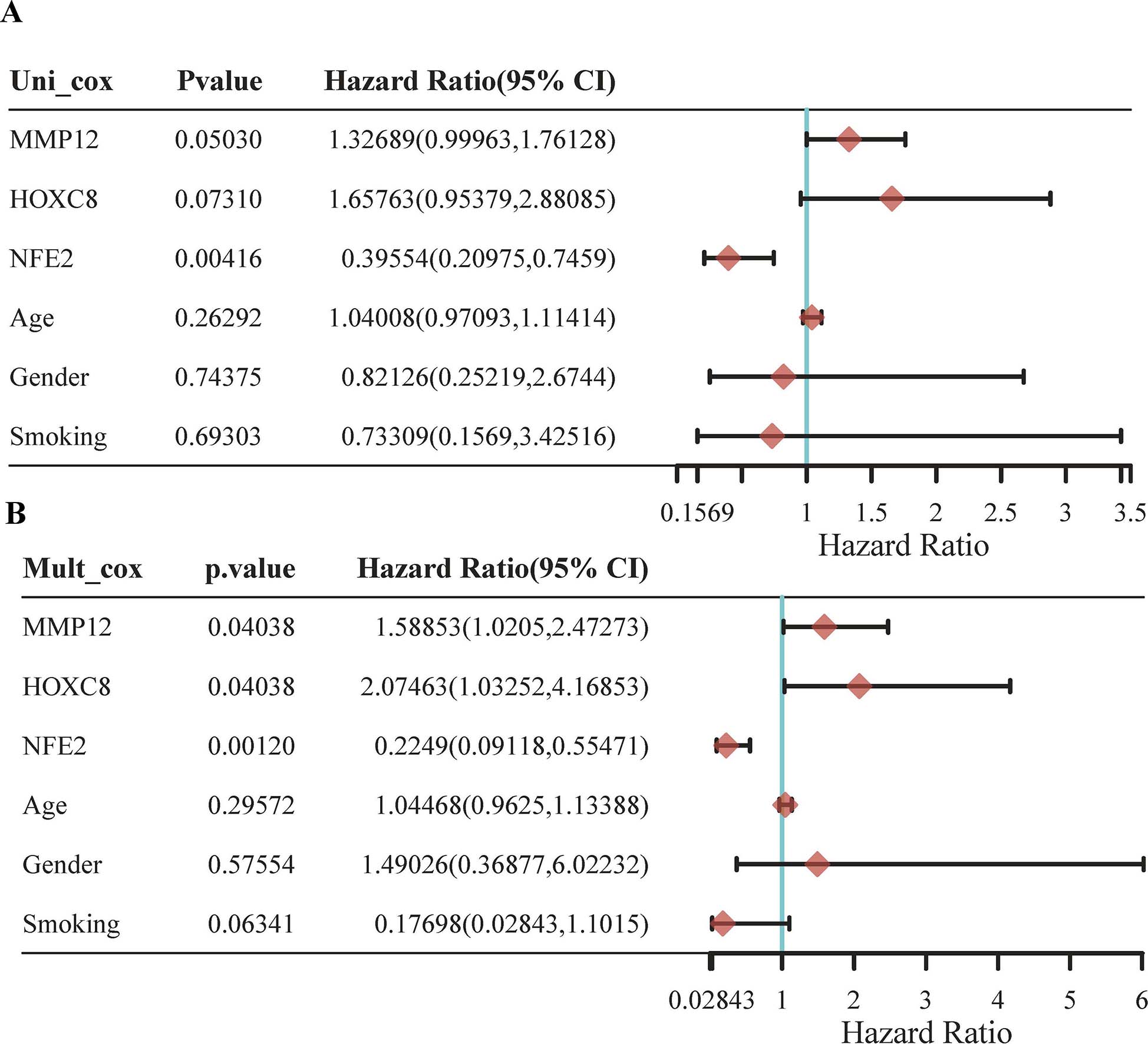

Supplement: Supplementary file 17 — Additional file 17. The figure of univariate and multivariate cox analysis of target genes expression and clinical characteristics. Figure S1. Univariate and multivariate cox analysis of target genes expression and clinical characteristics. A Univariate cox analysis of gene expression and clinical characteristics; B. multivariate cox analysis of gene expression and clinical characteristics. [file 12859_2023_5137_MOESM17_ESM.jpg]

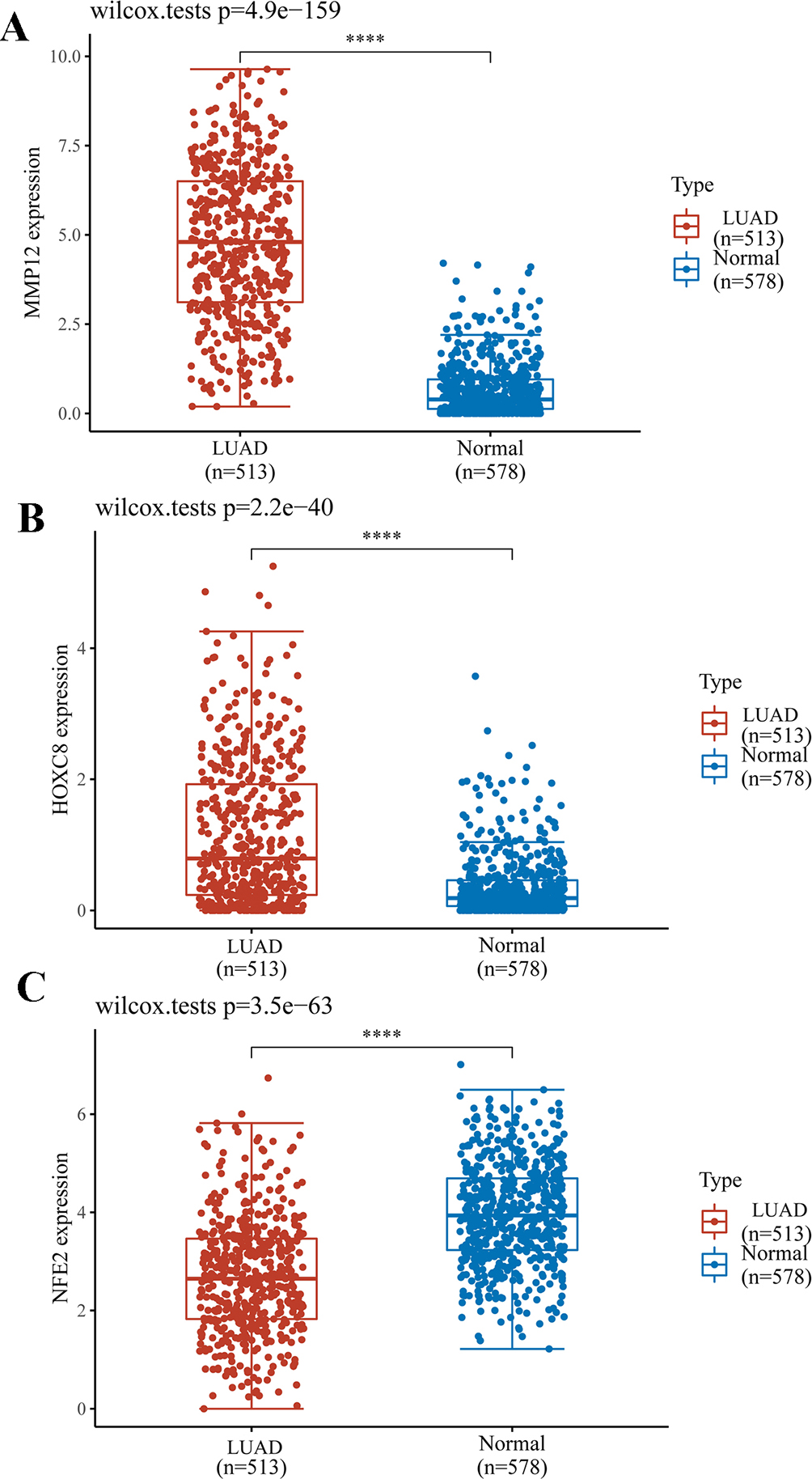

Supplement: Supplementary file 18 — Additional file 18.The figurge of differential expression distribution of target genes in all stages of tumors and adjacent tissues from HPA database. Figure S2. Differential expression distribution of target genes in all stages of tumors and adjacent tissues. A. Differential expression of MMP12; B. Differential expression of HOXC8; C. Differential expression of NFE2. [file 12859_2023_5137_MOESM18_ESM.jpg]

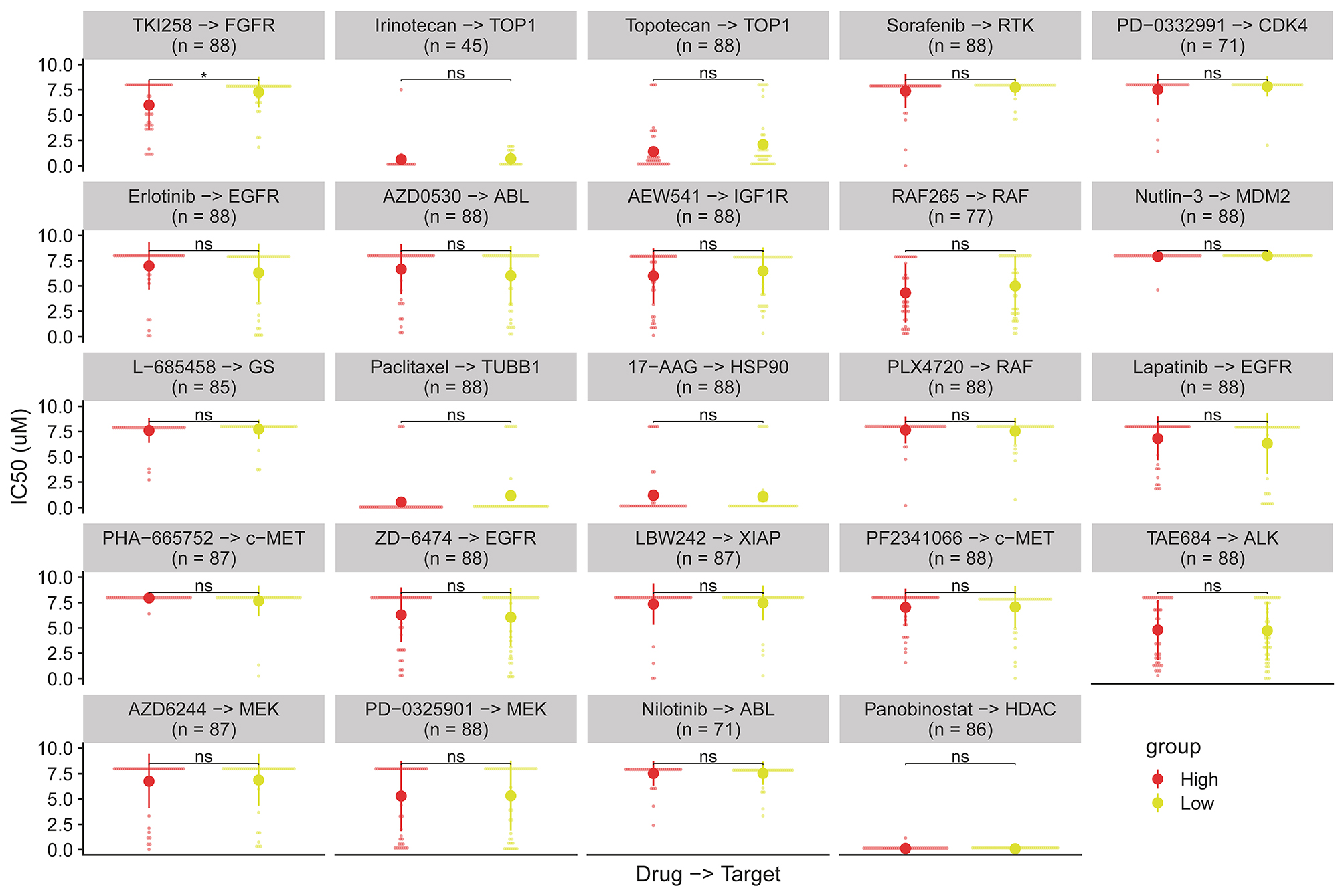

Supplement: Supplementary file 19 — Additional file 19.The drug sensitivity analysis of HOXC8 from CCLE database. Figure S3. Correlation bubble chart for drug sensitivity analysis of IC50 of different drugs in HOXC8 high and low groups from CCLE database.*p < 0.05, **p < 0.01, ***p < 0.001, asterisks (*) stand for significance levels. [file 12859_2023_5137_MOESM19_ESM.jpg]

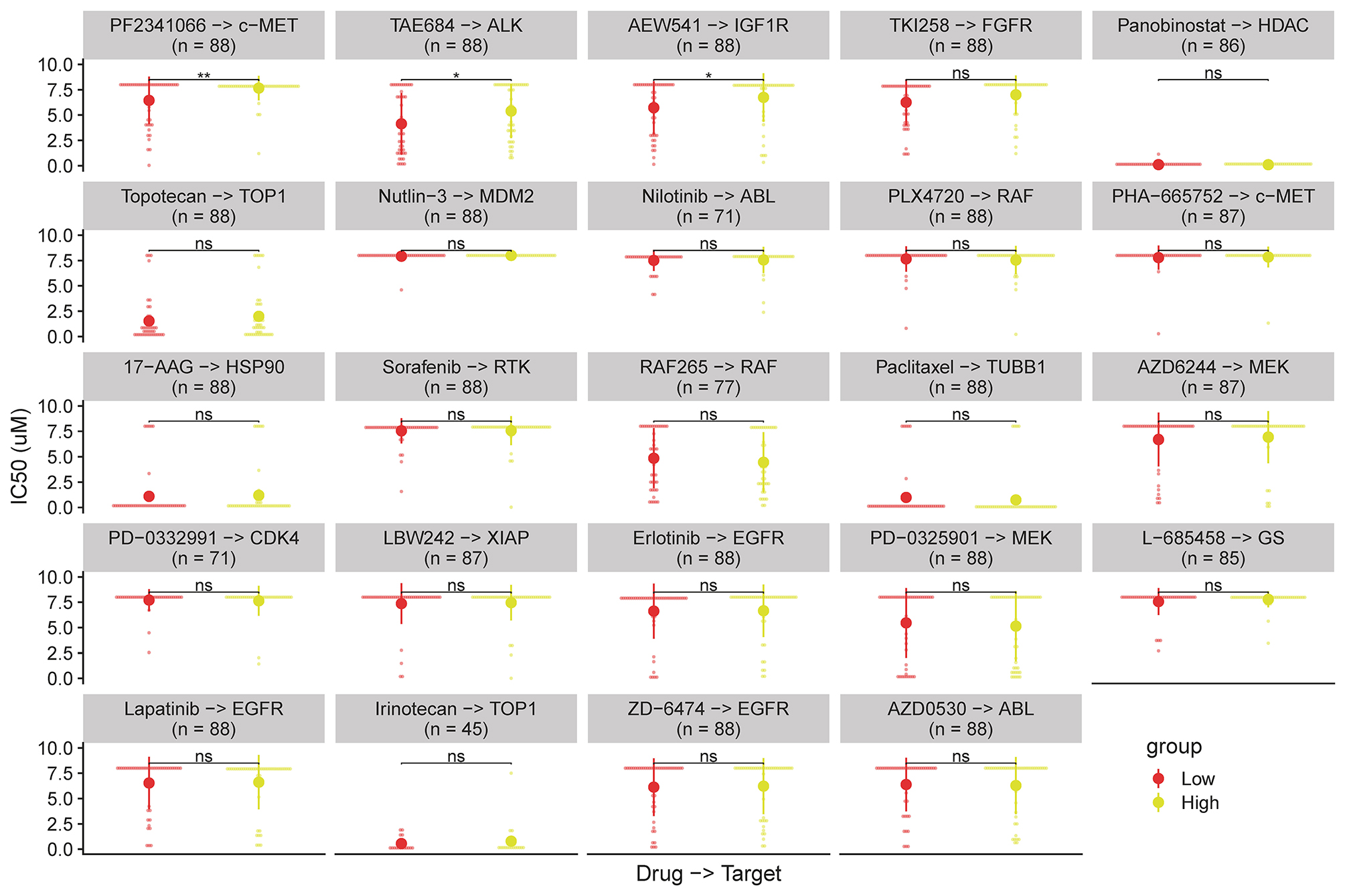

Supplement: Supplementary file 20 — Additional file 20. The drug sensitivity analysis of MMP12 from CCLE database. Figure S4. Correlation bubble chart for drug sensitivity analysis of IC50 of different drugs in MMP12 high and low groups from CCLE database. *p < 0.05, **p < 0.01, ***p < 0.001, asterisks (*) stand for significance levels. [file 12859_2023_5137_MOESM20_ESM.jpg]
